# Water Temperature and Practical Feeding Regimes Interact to Affect Growth, Enzyme Activities, and Histological Characteristics in Juvenile and Sub-Adult Chinese Mitten Crab (*Eriocheir sinensis*)

**DOI:** 10.3390/ani16193141

**Published:** 2026-10-07

**Authors:** Min Wei, Yuyan Sun, Xinran Yin, Yijin Zhu, Yansong Zhang, Yi Xu, Zhongwei Li, Chao Guo

**Affiliations:** 1 Co-Innovation Center of Jiangsu Marine Bioindustry Technology, Jiangsu Ocean University, Lianyungang 222005, China; sun010911@163.com (Y.S.); xinran_y@outlook.com (X.Y.); zhuyijin0910@163.com (Y.Z.); lucixzys@gmail.com (Y.Z.); m15163056667@163.com (Y.X.); 2 Jiangsu Key Laboratory of Marine Biotechnology, Jiangsu Ocean University, Lianyungang 222005, China; 3 Jiangsu Sihong County Jinshui Special Aquaculture Co., Ltd., Suqian 223900, China; li843174325@163.com; 4 State Key Laboratory of Freshwater Aquaculture Germplasm Innovation and Efficient Aquaculture, Institute of Hydrobiology, Chinese Academy of Sciences, Wuhan 430072, China; guochao@ihb.ac.cn

**Keywords:** *Eriocheir sinensis*, practical feeding regime, water temperature, antioxidant response, histopathology

## Abstract

The Chinese mitten crab (*Eriocheir sinensis*) is commonly cultured using compound feed or iced fresh fish, but its performance may vary with water temperature and developmental stage. This 28-day indoor study evaluated the interactive effects of feeding regime and water temperature on growth, pooled survival (reported descriptively), apparent feed disappearance, tissue enzyme activities, and qualitative intestinal and gill histology in juvenile and sub-adult *E. sinensis*. Crabs were assigned to compound-feed or iced-fresh-fish regimes at 20, 24, 28, or 32 °C. Significant feeding regime × temperature interactions were detected for growth traits in both developmental stages. Under the present experimental conditions, the highest numerical juvenile body-weight gain was observed under the compound-feed regime at 28 °C, whereas the highest numerical sub-adult body-weight gain was observed under the compound-feed regime at 24 °C. Apparent feed disappearance was generally higher in the iced-fresh-fish regime but did not consistently correspond to greater growth. Linear mixed-effects models revealed tissue- and stage-specific enzyme responses, including significant feeding regime × temperature × time interactions for selected metabolic, ion-transport, and immune-related activities. Qualitative histological observations showed treatment-associated structural changes in the intestine and gill. Because the two regimes differed fundamentally in moisture content and nutrient density, our results reflect responses to commercial feeding practices rather than an evaluation of nutritionally equivalent diets.

## Simple Summary

Chinese mitten crab (*Eriocheir sinensis*) is widely farmed using either commercial pellets or iced fresh fish, but the effectiveness of these feeding practices may vary with water temperature and developmental stage. We conducted a 28-day indoor experiment to examine the responses of juvenile and sub-adult crabs to the two feeding regimes at 20, 24, 28, and 32 °C. Growth, survival, apparent feed disappearance, tissue enzyme activities, and intestinal and gill histology were evaluated. The results showed that feeding responses depended on both temperature and developmental stage. Pellet-fed juveniles showed the highest numerical growth at 28 °C, whereas sub-adults showed the highest numerical body-weight gain at 24 °C. Although iced fresh fish generally showed higher apparent feed disappearance values, this did not consistently result in greater growth, highlighting the need to distinguish apparent feed disappearance from actual feed intake. Sub-adult survival was low at 32 °C. Because tissue measurements were obtained only from crabs alive at sampling, these findings may not represent the condition of the initially stocked population.

## 1. Introduction

The Chinese mitten crab (*Eriocheir sinensis*) is one of the most economically important cultured crustaceans in China [1]. The juvenile and sub-adult stages represent important periods of development because growth, molting, feeding activity and physiological responses during these stages strongly influence subsequent performance under culture conditions [2,3]. However, biological responses during these developmental stages may differ because body size, metabolic demand, and physiological status change substantially during development. In commercial culture, crabs are commonly provided with either compound feed or iced fresh fish. Nutritional requirements of *E. sinensis* have been investigated to determine several key nutrients. Dietary protein requirements vary with developmental stage and sex, with approximately 34.8% reported for maximal growth in juveniles and 38.5% for female and at least 42.0% for male sub-adults [4,5,6]. Optimal dietary lipid levels in juveniles vary with salinity, ranging from approximately 5 to 13% [7]. In addition, dietary requirements of essential amino acids, including approximately 2.0% arginine and 2.55% lysine, have been reported for juveniles [8]. These feeding practices differ not only in physical characteristics and storage requirements but also in moisture content, nutrient density, and water stability. Compound feed is easier to standardize and manage, whereas iced fresh fish is highly palatable and is still used in some farming systems [9,10]. Previous studies have shown that different feeding practices can lead to different intestinal microbial and metabolic profiles in *E. sinensis* [11]. Dietary regulation may affect growth and several physiological indicators, including digestive activity, antioxidant responses, and intestinal condition [12]. However, compound feed and iced fresh fish should not be regarded as nutritionally equivalent diets when they differ substantially in moisture and nutrient composition. Therefore, comparisons between these treatments are more appropriately interpreted as comparisons between practical feeding regimes rather than strictly controlled dietary formulations.

Water temperature is another key environmental factor influencing the biology of cultured crustaceans. As an ectothermic species, *E. sinensis* adjusts feeding, metabolism, molting and immune responses according to ambient temperature. Within a suitable thermal range, increasing temperature may promote feeding and growth, whereas excessive temperature can disturb physiological homeostasis and reduce survival or growth performance. Previous studies have generally suggested that approximately 20–28 °C represents a suitable thermal range for growth and feeding of *E. sinensis*, although the reported optimum may vary with developmental stage and the physiological trait evaluated [13,14,15]. For example, early juvenile *E. sinensis* showed faster growth and molting at 28–30 °C than at lower temperatures under controlled laboratory conditions [13], whereas other studies have reported broader suitable ranges of approximately 20–28 °C or 23–30 °C for juvenile growth [14,15]. Recent studies further indicate that heat stress can induce oxidative and immune disturbances and alter intestinal homeostasis in juvenile *E. sinensis*. Chronic exposure to high temperature, such as 30 °C compared with 24 °C, has been associated with reduced survival, disruption of antioxidant defenses, and increased oxidative stress [16,17]. Heat stress has also been reported to alter immune-related enzyme activities and the diversity and structure of the intestinal microbiota [18]. In addition, prolonged high-temperature exposure may compromise intestinal integrity, whereas dietary interventions can partially alleviate these adverse responses [17]. These findings indicate that thermal stress can affect multiple physiological systems in juvenile *E. sinensis*, rather than growth alone. Transcriptomic evidence from gill and hepatopancreas tissues also suggests that these organs are closely involved in thermal adaptation in *E. sinensis* [19]. Consequently, responses to feeding practices may vary across water temperatures and may also differ between developmental stages.

Growth performance alone cannot fully describe how animals respond to feeding and thermal conditions. Digestive enzymes provide information on biochemical processes associated with nutrient digestion, whereas antioxidant, metabolic, and immune-related enzymes can reflect changes in tissue biochemical responses under different environmental conditions [12,15,20]. In addition, the intestine and gill are closely associated with digestion, barrier function, respiration and ion regulation. Histological observations of these tissues can provide descriptive information regarding treatment-associated changes in tissue structure [21,22]. Combining growth measurements with apparent feed disappearance, tissue enzyme activity and qualitative histological observations may therefore provide a broader assessment of the responses of juvenile and sub-adult *E. sinensis* to practical feeding regimes across different thermal conditions.

The present study examined the combined effects of two practical feeding regimes, compound feed and iced fresh fish, and four water temperatures (20, 24, 28, and 32 °C) on growth, pooled survival, apparent feed disappearance, tissue enzyme activities, and qualitative intestinal and gill histology in juvenile and sub-adult *E. sinensis* during a 28-day indoor experiment. We hypothesized that (i) growth and tissue biochemical responses would be influenced by interactions between feeding regime and water temperature; (ii) these responses would differ between juvenile and sub-adult crabs; and (iii) higher apparent feed disappearance in the iced-fresh-fish regime would not necessarily correspond to greater growth. Because the two feeding regimes differed in moisture and nutrient characteristics, this study was designed to evaluate responses to practical feeding practices rather than to compare nutritionally equivalent diets.

## 2. Materials and Methods

### 2.1. Experimental Design

Juvenile and sub-adult *E. sinensis* were obtained from a culture base in Hongze Lake, Sihong County, Jiangsu Province, China. Because the juvenile and sub-adult crabs had not yet reached full sexual development, individuals were not sexed before allocation to the experimental treatments. Before the experiment, crabs that were actively molting, had recently completed molting, or exhibited soft shells were excluded. Only intact and healthy individuals without apparent molting activity were selected for the experiment. Before the formal experiment, the crabs underwent a one-week laboratory acclimation period. During this period, crabs were gradually acclimated to their assigned experimental water temperature and feeding regime before the 28-day experimental culture period began.

At the beginning of acclimation, the ambient water temperature was approximately 28 °C. For treatments below 28 °C, the environmental temperature was gradually reduced using air conditioning, while water temperature was adjusted accordingly at a rate of approximately 2 °C per day. For treatments above 28 °C, water temperature was increased using thermostatic heating rods (Sensen Group Co., Ltd., Zhoushan, China) at approximately 1 °C per day. The lower warming rate was used as a precaution to minimize the potential for acute thermal stress during exposure to temperatures above the initial acclimation temperature, whereas the slightly faster cooling rate shortened the transition period while maintaining a gradual temperature change. The target temperature was maintained for at least 3 days before the formal 28-day experiment began.

The experiment used a two-factor design with practical feeding regime and water temperature as fixed factors. The two practical feeding regimes were compound feed and iced fresh fish, and the four water temperatures were 20, 24, 28 and 32 °C. The experiment followed a completely randomized practical feeding regime × water temperature factorial design with three independent replicate tanks per treatment combination.

The experimental rearing tanks were made of polyethylene (PE), with dimensions of 75.5 cm × 55.0 cm × 45.5 cm (length × width × height) and a working volume of 160 L. Each tank operated as a physically discrete unit without shared water recirculation. Water temperature was independently maintained in each tank using a dedicated submersible digital thermostatic heater (Sensen Group Co., Ltd., Zhoushan, China). Continuous aeration was provided using an individual air stone. Tap water was used as the culture water and was aerated with air stones for at least 48 h before use, with the water exposed to natural sunlight during this period. No salt was added, and no biological filtration system was used. An artificial shrimp shelter was provided in each tank to allow crabs to seek refuge.

A photoperiod of 16h light:8h dark (16L:8D) was maintained throughout the acclimation and experimental periods. Experimental tanks were randomly positioned within the indoor rearing facility to minimize potential spatial effects.

Immediately before the start of the formal 28-day culture experiment, baseline samples were collected after completion of the one-week acclimation period. These samples are referred to as week 0 samples throughout the manuscript. Thus, week 0 represents the physiological and morphological status of crabs immediately before the 28-day experimental culture period following acclimation to the assigned temperature and feeding regime.

Crabs sampled from the same tank were treated as subsamples, and measurements obtained from individuals within each tank were averaged before statistical analysis. Therefore, the individual tank was considered the experimental unit (*n* = 3 replicate tanks per treatment combination).

A total of 720 juvenile crabs (initial body weight 13.03 ± 0.26 g; carapace length 2.80 ± 0.02 cm; carapace width 3.06 ± 0.02 cm) were used, with 30 crabs per replicate tank. A total of 360 sub-adult crabs (initial body weight 57.32 ± 0.88 g; carapace length 4.79 ± 0.03 cm; carapace width 5.17 ± 0.03 cm) were used, with 15 crabs per replicate tank. Because the crabs were obtained from a commercial culture base, their exact age or days post-settlement were not available. The juvenile and sub-adult stages were therefore identified according to the developmental stage classification used for the experimental animals at the source farm.

To assess practical feeding strategies commonly adopted in aquaculture, two distinct feeding regimes were evaluated: a commercial formulated extruded diet (Jiangsu Haipuri Feed Co., Ltd., Xinghua, Jiangsu, China) and iced fresh fish. Because this study aimed to compare practical farming regimes rather than isonitrogenous/isocaloric nutrient variables, the proximate composition of the diets was characterized based on manufacturer-guaranteed specifications for the commercial feed and the established literature values for the fresh fish (Table 1) [23]. On a dry-matter (DM) basis, the compound feed contained at least 42.0% (juvenile) and 36.0% (sub-adult) crude protein, whereas the iced fresh fish, obtained from a local aquatic product supplier in Sihong County, Jiangsu Province, China, and consisting predominantly of *Collichthys lucidus*, contained approximately 62.50 ± 2.18% crude protein and 20.22 ± 1.10% crude lipid on a DM basis, despite its high moisture content.

### 2.2. Feeding and Aquaculture Management

Crabs were fed once daily at 16:00. Daily feed rations were provided on an as-fed (wet weight) basis at approximately 1.0–2.0% of the total live body weight in each tank. The feeding level was determined based on preliminary feeding observations to reduce excessive feed residues and minimize deterioration of water quality. The feeding amount was adjusted every two weeks according to changes in crab biomass determined by weighing, and dead individuals were excluded from subsequent biomass calculations. The same feeding-rate range was applied across all temperature treatments and developmental stages. This approach was used to reflect the practical feeding conditions of the two feed types, which were supplied in their respective commercial feeding forms rather than on an equal dry-matter or nutrient basis.

Throughout the 28-day experiment, all tanks were monitored daily and dead crabs were removed when observed. Crabs removed according to the scheduled sampling design were not classified as mortalities.

Uneaten feed was collected immediately before the next day’s scheduled feeding at 16:00, approximately 24 h after the previous feeding, to estimate of the apparent daily feed disappearance rate (ADFR). Tanks were continuously aerated, dissolved oxygen was maintained above 7.0 mg/L, and pH ranged from 7.0 to 8.0. Ammonia and nitrite concentrations were not routinely monitored during the experiment. In order to maintain water quality and remove residual feed and fecal matter, fifty percent of the water was exchanged daily with fresh water that had been pre-aerated and adjusted to the target temperature. Because the compound feed and iced fresh fish differed substantially in moisture content, nutrient composition and physical characteristics, feeding quantities were controlled on an as-fed basis to reflect practical feeding conditions. Therefore, the two treatments were regarded as practical feeding regimes rather than nutritionally equivalent diets, and the present study was not designed to compare the biological effects of the two feeds under equal dry-matter or nutrient intake.

### 2.3. Growth Performance and Survival

At the beginning and end of the trial, body weight, carapace length and carapace width were measured. Measurements from individuals within the same replicate tank were averaged before calculation of tank-level growth responses.

Absolute changes in body weight, carapace length, and carapace width were calculated from the difference between the final and initial measurements. Molting was not continuously monitored or individually tracked during the experimental period; therefore, the precise stage of molting at the time of the initial and final morphometric measurements was not recorded. In addition, relative growth indices were calculated to facilitate comparison of growth responses among treatment groups with different initial body size.

Weight gain rate (WGR) was calculated as:

WGR (%) = [(Wf − Wi)/Wi] × 100

where Wi and Wf represent the initial and final body weights, respectively.

Specific growth rate (SGR) was calculated as:

SGR (%) = [ln(Wf) − ln(Wi)] × 100/t

where t represents the duration of the experiment in days.

Relative changes in carapace length and carapace width were calculated as:

Relative carapace length change (%) = [(CLf − CLi)/CLi] × 100

Relative carapace width change (%) = [(CWf − CWi)/CWi] × 100

where CLi and CLf represent the initial and final carapace lengths, respectively, and CWi and CWf represent the initial and final carapace widths, respectively.

For survival calculation, crabs removed according to the scheduled sampling design were excluded from mortality counts. Dead crabs were removed when observed during the experiment, but daily mortality was not recorded. At the end of the experiment, surviving crabs from the three replicate tanks within each treatment were pooled and counted, and the survival percentage was calculated based on the final number of surviving crabs relative to the total number initially stocked. Therefore, survival was summarized at the treatment level, and no inferential statistical analysis was performed.

### 2.4. Apparent Daily Feed Disappearance Rate

During the 28-day experimental period, residual feed was collected daily by siphoning immediately before the next feeding, approximately 24 h after feed provision; gently rinsed with distilled water; and dried to constant weight at 60 °C. The physical condition of the residual feed was not systematically assessed, and therefore its integrity after the feeding interval was not quantified. Apparent daily feed disappearance rate (ADFR) was calculated as:

ADFR (%) = [(W2 − W1)/W0] × 100

where W2 is the daily amount of feed supplied on an as-fed (wet-weight) basis, W1 is the recovered uneaten feed after rinsing and drying to constant weight at 60 °C, and W0 is the initial total body weight of crabs in the tank. Because feed supply and recovered residual feed were determined on different weight bases, ADFR was considered an apparent feed disappearance index rather than a direct measure of feed intake.

ADFR was calculated on an as-fed basis and was defined as apparent feed disappearance rather than true voluntary feed intake. In particular, iced fresh fish may soften, fragment, disintegrate, and release soluble components during immersion and handling, potentially affecting recovery of residual feed. Therefore, ADFR was interpreted as an apparent feeding index rather than a precise estimate of actual dry-matter intake.

### 2.5. Tissue Sampling and Enzyme Assays

At weeks 0, 2 and 4, three crabs were sampled from each replicate tank. Hepatopancreas, intestine, gill and muscle tissues were collected, placed in cryovials and stored at -80 °C until analysis. For statistical analysis, tissue data from crabs sampled from the same tank were averaged so that the tank remained the experimental unit.

Sampled tissues were accurately weighed and homogenized on ice in 9 volumes (*w*/*v*, 1:9) of ice-cold phosphate-buffered saline (PBS). Tissue homogenates were centrifuged at 2500× *g* for 10 min at 4 °C using a Centrifuge 5425 R (Eppendorf SE, Hamburg, Germany), and the resulting supernatants were separated and stored at 4 °C for enzyme assays. Total soluble protein content in each supernatant was quantified using a bicinchoninic acid (BCA) protein assay kit (Nanjing Jiancheng Bioengineering Institute, Nanjing, China; Cat. No. A045-4-2) at λ = 595 nm.

All enzymatic assays were conducted using commercial diagnostic kits (Nanjing Jiancheng Bioengineering Institute, Nanjing, China) following manufacturer-specified reaction chemistries. Digestive enzymes, including amylase (AMS; Cat. No. C016-1-2; iodine–starch chromogenic method, λ = 660 nm) and lipase (LPS; Cat. No. A054-2-1; methyl haloperidol substrate method, λ = 580 nm), were assayed from hepatopancreatic and intestine supernatants. Superoxide dismutase (SOD; Cat. No. A001-3; WST-1 method, λ = 450 nm) was quantified in the hepatopancreas, intestine, gill and muscle tissues and lactate dehydrogenase (LDH; Cat. No. A020-2-2; 2,4-dinitrophenylhydrazine colorimetric method, λ = 440 nm) was quantified in the hepatopancreas, gill and muscle tissues to evaluate antioxidant capacity and glycolytic metabolism, respectively. Na^+^/K^+^-ATPase and Ca^2+^/Mg^2+^-ATPase (Cat. No. A016-2) activities were measured in hepatopancreatic and muscle homogenates as indicators of energy-dependent ion-transport-related processes, based on the enzymatic liberation of inorganic phosphate (Pi) at λ = 660 nm. Phenoloxidase (PO; Cat. No. H247-1-2; L-DOPA oxidation assay, λ = 450 nm) was determined from the hepatopancreas and gill.

All enzyme activities were normalized directly to tissue soluble protein concentration and expressed as specific activities. All assays were performed in technical triplicate using an ELISA microplate reader (Thermo Fisher Scientific (Shanghai) Instruments Co., Ltd., Shanghai, China). All samples were anonymized and randomized using alphanumeric codes prior to measurement.

### 2.6. Histological Analysis

At weeks 0 and 4, intestine and gill tissues were collected from three crabs per replicate tank. For each crab, three consecutive intestinal sections and three consecutive gill sections were examined microscopically. Tissue sections from all three sampled crabs in each of the three replicate tanks were examined, corresponding to nine crabs per feeding regime × temperature combination, developmental stage, and sampling time. Selected micrographs are presented to illustrate the observed morphological features. Histological observations were descriptive, and no quantitative histomorphometry or standardized lesion scoring was performed. Samples were rinsed with physiological saline and fixed in 4% paraformaldehyde for 24 h. The tissues were dehydrated through graded ethanol (75%, 85%, 95%, 100%, and 100%), cleared in xylene, embedded in paraffin and sectioned at 5 μm. Sections were stained with hematoxylin and eosin and observed using a Nikon 90i microscope (Nikon Corporation, Tokyo, Japan). Histological descriptions were based on representative sections from each treatment. No morphometric measurements or statistical severity scoring was conducted, and histological observations are interpreted strictly in a descriptive context.

### 2.7. Data Analysis

Data are presented as mean ± standard error (SE). Each tank was considered the experimental unit. For each sampling point, values from crabs sampled within the same tank were averaged before statistical analysis.

Initial growth-related measurements obtained immediately before the formal 28-day culture period (week 0) were first examined to determine whether body weight, carapace length, and carapace width differed among the feeding regime × temperature combinations following the one-week acclimation period. Growth responses during the 28-day culture period were evaluated separately for juvenile and sub-adult crabs using two-way analysis of variance (ANOVA), with practical feeding regime and water temperature as fixed factors. Because initial body size differed among treatment combinations, relative growth indices, including weight gain rate and specific growth rate, were calculated in addition to absolute growth increments to facilitate comparison among treatments.

Pooled survival was presented descriptively because replicate-level survival data were not available for inferential statistical analysis. Survival values therefore were not subjected to ANOVA or other inferential statistical comparisons.

ADFR and enzyme activities were analyzed separately for juvenile and sub-adult *E. sinensis* using linear mixed-effects models. Feeding regime, water temperature, time, and their two- and three-way interactions were specified as fixed effects. Tank was specified as the subject of repeated measurements and included as a random intercept to account for the dependence among observations obtained from the same tank across times. A diagonal covariance structure was applied to model within-tank repeated observations over time. Different crabs were sampled at each time point, and repeated observations were therefore defined at the tank level rather than at the individual level. All planned tank-level observations included in the analyses were available, and no data imputation was performed. Mortality occurring during the experiment was not treated as a missing observation or imputed value; dead crabs were removed when observed, and the physiological measurements at each sampling point were based on the surviving crabs available for scheduled sampling.

Because week 0 samples were collected after the one-week acclimation period but immediately before the formal 28-day experimental culture period, week 0 was treated as a post-acclimation baseline sampling point. The changes observed from week 0 to week 4 are a normal phenomenon occurring during the aquaculture experiment, rather than a stress response exhibited by the experimental animals to an unfamiliar environment.

Higher-order interactions were evaluated before interpreting lower-order effects. When significant interactions occurred, interpretation focused on condition-specific simple effects rather than lower-order main effects alone, with Bonferroni-adjusted comparisons performed within each relevant time or temperature condition. Model assumptions were assessed by examining residual distributions and residual plots. No substantial deviations from normality or heteroscedasticity were detected. Statistical significance was set at *p* < 0.05. Given the three replicate tanks per treatment, the power to detect small effects, particularly higher-order interactions, may have been limited. Therefore, non-significant interaction effects were interpreted cautiously. Analyses were performed using IBM SPSS Statistics 27.0. Graphs were prepared using Origin 2021. Complete fixed-effect tests are provided in the Supplementary Materials.

### 2.8. Animal Welfare and Experimental Refinement

The animal study protocol was approved by the Laboratory Animal Ethics Committee of Jiangsu Ocean University (approval no. 20260003; date of approval: 5 January 2026). All procedures followed institutional guidelines for the care and use of experimental aquatic animals. Crabs were inspected daily; dead or moribund animals were removed immediately. No surgical procedures were performed.

## 3. Results

### 3.1. Effects of Different Feeds and Temperatures on Growth Traits of E. sinensis

After the 28-day culture trial, pooled survival rates varied among feeding regime–temperature treatments in both juvenile and sub-adult *E. sinensis* (Table 2). In juveniles fed compound feed, survival decreased from 75.56% at 20 °C to 58.89% at 28–32 °C. In the iced-fresh-fish groups, juvenile survival fluctuated across temperatures, with the lowest value observed at 28 °C. In sub-adults, survival remained high in the compound-feed groups at 20–28 °C, ranging from 82.22% to 84.44%, but decreased markedly to 37.78% at 32 °C. Sub-adults fed iced fresh fish showed lower pooled survival at 20–28 °C and the lowest survival at 32 °C, where 31.11% of crabs survived.

**Figure 1:**
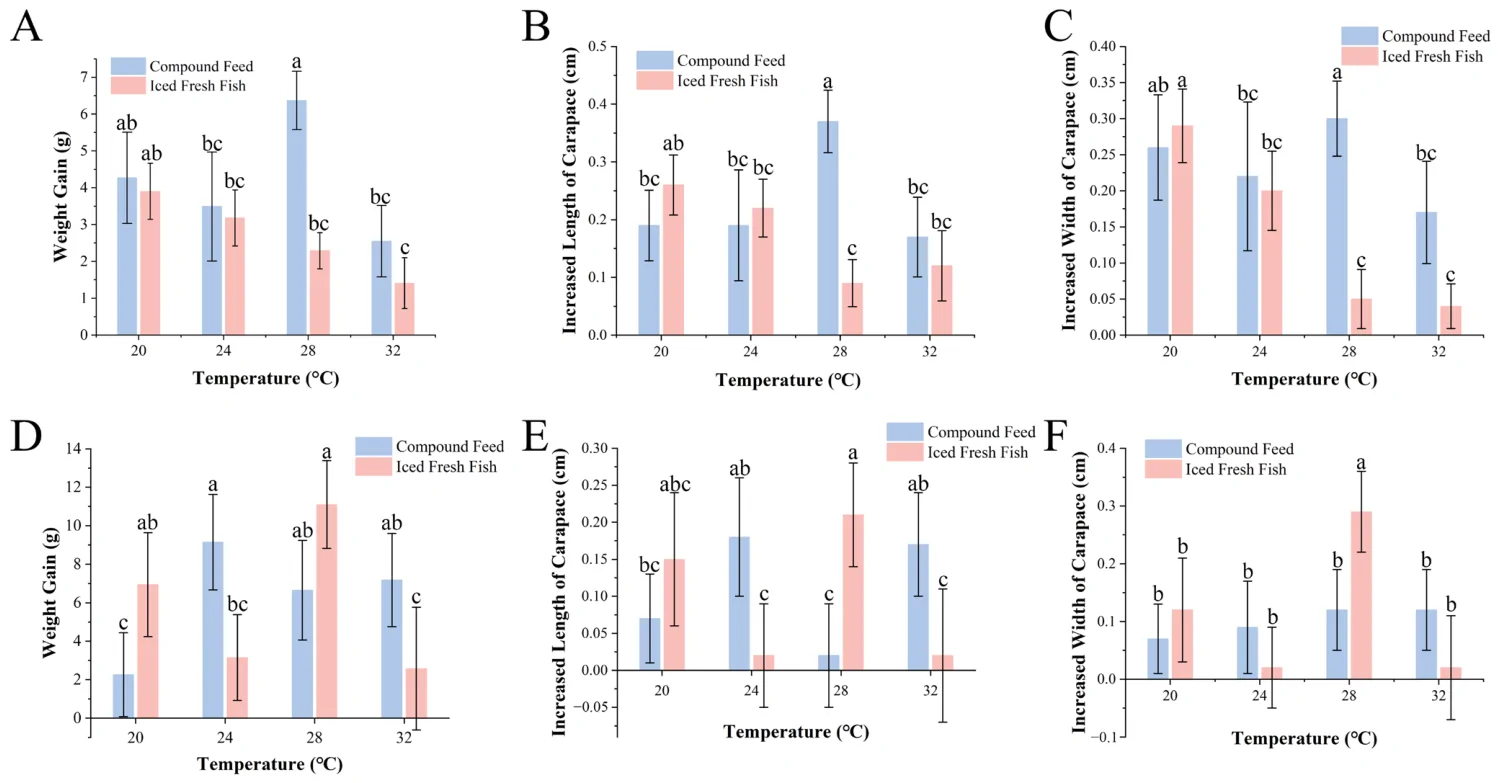
Effects of feeding regime and water temperature on growth increments of juvenile and sub-adult Chinese mitten crab (*Eriocheir sinensis*) after the 4-week culture period. (**A**–**C**) represent body-weight gain, carapace length increment and carapace width increment in juvenile crabs, respectively. (**D**–**F**) represent body-weight gain, carapace length increment and carapace width increment in sub-adult crabs, respectively. Data are expressed as mean ± SE (*n* = 3 replicate tanks). The effects of feeding regime, water temperature and their interaction were tested using two-way ANOVA. Detailed fixed-effect results are reported in Table S5. Within each panel, lowercase letters indicate the results of Bonferroni-adjusted pairwise comparisons among feeding regime × temperature combinations. Bars sharing at least one letter are not significantly different, whereas bars with no letters in common differ significantly at *p* < 0.05.

The effects of feeding regime and water temperature on the growth performance of juvenile and sub-adult *E. sinensis* are presented in Tables S1–S4 and Figure 1. Initial body weight, carapace length, and carapace width differed among some treatment combinations (Tables S1 and S3). These variations likely reflected natural individual heterogeneity despite random allocation. Two-way ANOVA revealed significant feeding regime × temperature interactions for body-weight gain, carapace length increment, and carapace width increment in both developmental stages (*p* = 0.001–0.027; Table S5). Therefore, growth responses were evaluated according to specific feeding regime–temperature combinations rather than by considering the main effects alone.

In juvenile *E. sinensis*, growth performance differed markedly among feeding regime–temperature combinations. Under the compound-feed regime, WGR increased from 25.85 ± 8.10% at 20 °C to 46.76 ± 7.79% at 28 °C, with a corresponding increase in SGR from 0.82 ± 0.23 to 1.37 ± 0.19% day^−1^. Relative carapace length change was 13.03 ± 2.11% at 28 °C, significantly higher than the 6.15 ± 1.89% observed at 20 °C. Relative carapace width change was numerically greatest at 28 °C (9.65 ± 1.79%), but did not differ significantly among the four temperature treatments (Table S2). Growth performance subsequently declined at 32 °C, where WGR and SGR decreased to 21.13 ± 8.63% and 0.68 ± 0.25% day^−1^, respectively. In contrast, the iced-fresh-fish regime showed a different pattern, with WGR and SGR generally decreasing as temperature increased. WGR declined from 30.90 ± 12.98% at 20 °C to 14.86 ± 7.95% at 32 °C, while SGR decreased from 0.96 ± 0.35 to 0.49 ± 0.25% day^−1^. Relative carapace length and width changes were also substantially lower at 28 and 32 °C than at 20 °C. At 28 °C, juveniles receiving compound feed exhibited greater body-weight gain, SGR, relative carapace length change, and relative carapace width change than those receiving iced fresh fish (Table S2; Figure 1A–C).

In sub-adult *E. sinensis*, the response pattern differed from that of juveniles. Under the compound-feed regime, WGR and SGR were highest at 24 °C, reaching 16.20 ± 6.27% and 0.54 ± 0.19% day^−1^, respectively. Relative carapace length change was also highest at 24 °C, whereas relative carapace width change varied less markedly among temperatures. In the iced-fresh-fish regime, WGR and SGR were highest at 28 °C, reaching 21.68 ± 6.64% and 0.70 ± 0.19% day^−1^, respectively. Relative carapace length and width changes also reached their highest values at 28 °C, reaching 4.51 ± 2.17% and 5.82 ± 1.91%, respectively. At 20 °C, iced fresh fish resulted in higher WGR and SGR than compound feed, whereas at 24 °C the compound-feed regime produced higher WGR and SGR. At 32 °C, growth performance under the iced-fresh-fish regime was comparatively low, with a WGR and SGR of only 4.68 ± 6.28% and 0.16 ± 0.21% day^−1^, respectively (Table S4; Figure 1D–F).

### 3.2. Effects of Feeding Regime and Water Temperature on Apparent Daily Feed Disappearance Rate

**Figure 2:**
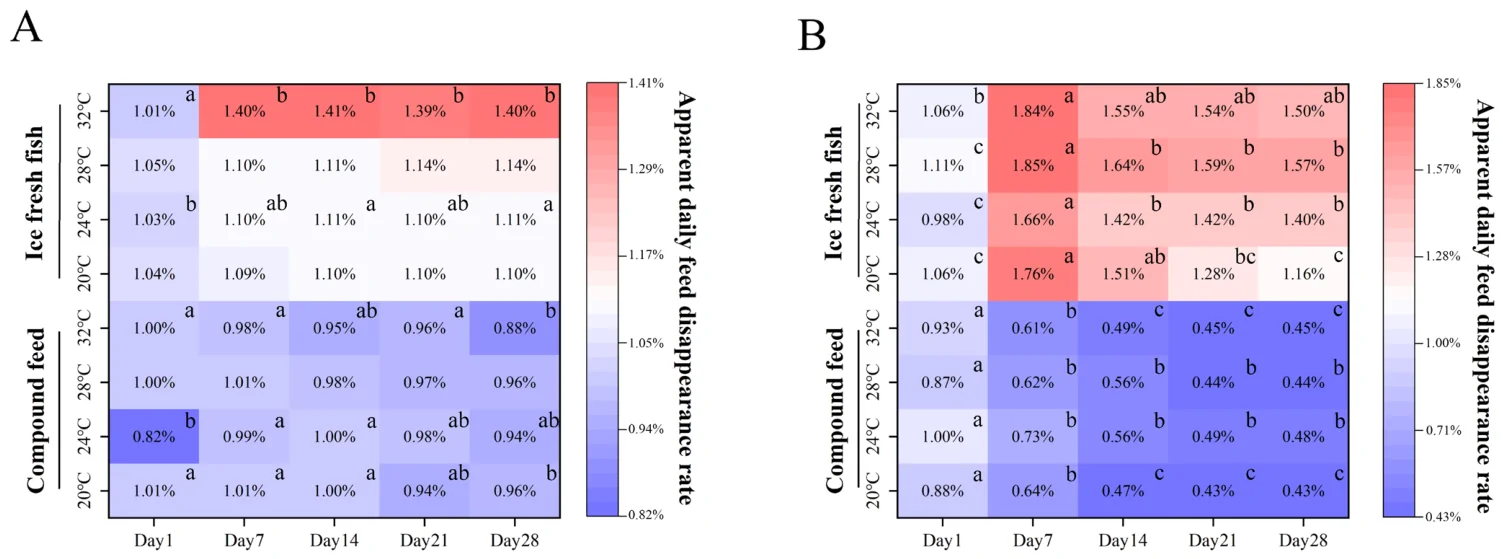
Heatmap of ADFR of juvenile and sub-adult Chinese mitten crab (*Eriocheir sinensis*) during the 28-day culture trial. (**A**,**B**) represent juvenile and sub-adult crabs, respectively. Values represent the means of three replicate tanks. ADFR was analyzed using a linear mixed-effects model with feeding regime, water temperature, time and their interactions as fixed effects, and tank as a random effect. Different lowercase letters within the same feeding regime × temperature combination indicate significant differences among time points (Bonferroni-adjusted simple-effect comparisons, *p* < 0.05).

The apparent daily feed disappearance rate (ADFR) of juvenile and sub-adult *E. sinensis* varied over the 28-day culture period. Within the same feeding regime × temperature combination, significant differences in ADFR among time points were indicated by different lowercase letters in Figure 2. Because ADFR was calculated from the difference between the feed supplied and residual feed recovered from each tank, it represents apparent feed disappearance rather than confirmed voluntary feed intake.

In juvenile *E. sinensis* (Figure 2A), ADFR was generally higher under the iced-fresh-fish regime than under the compound-feed regime. In the iced-fresh-fish groups, ADFR at 32 °C increased from approximately 1.01% on day 1 to about 1.40% on day 7 and subsequently remained at a relatively high level through the later culture period. In contrast, ADFR under the compound-feed regime was generally lower and showed a gradual decline during the culture period.

In sub-adult *E. sinensis* (Figure 2B), ADFR also showed clear temporal variation between feeding regimes. The iced-fresh-fish regime generally resulted in higher apparent feed disappearance than the compound-feed regime. Under iced fresh fish, ADFR increased during the early culture period and reached relatively high levels around day 7, particularly at 28 and 32 °C. During the middle and late stages, ADFR under the higher-temperature treatments remained relatively higher than that observed at 20 °C. In contrast, ADFR under the compound-feed regime was relatively high at the beginning of the experiment and subsequently declined after day 7.

Linear mixed-effects model analysis showed significant effects of feeding regime, water temperature, and time on ADFR in juvenile crabs (all *p* < 0.001; Table S6). Significant feeding regime × temperature, feeding regime × time, water temperature × time, and feeding regime × temperature × time interactions were also detected (all *p* < 0.001). In sub-adults, significant effects of feeding regime and time were detected (both *p* < 0.001), whereas the overall effect of water temperature was not significant (*p* = 0.655). The feeding regime × temperature interaction was also not significant (*p* = 0.450), but significant feeding regime × time, water temperature × time, and feeding regime × temperature × time interactions were observed (all *p* < 0.001; Table S6).

### 3.3. Digestive Enzyme Activity

**Figure 3:**
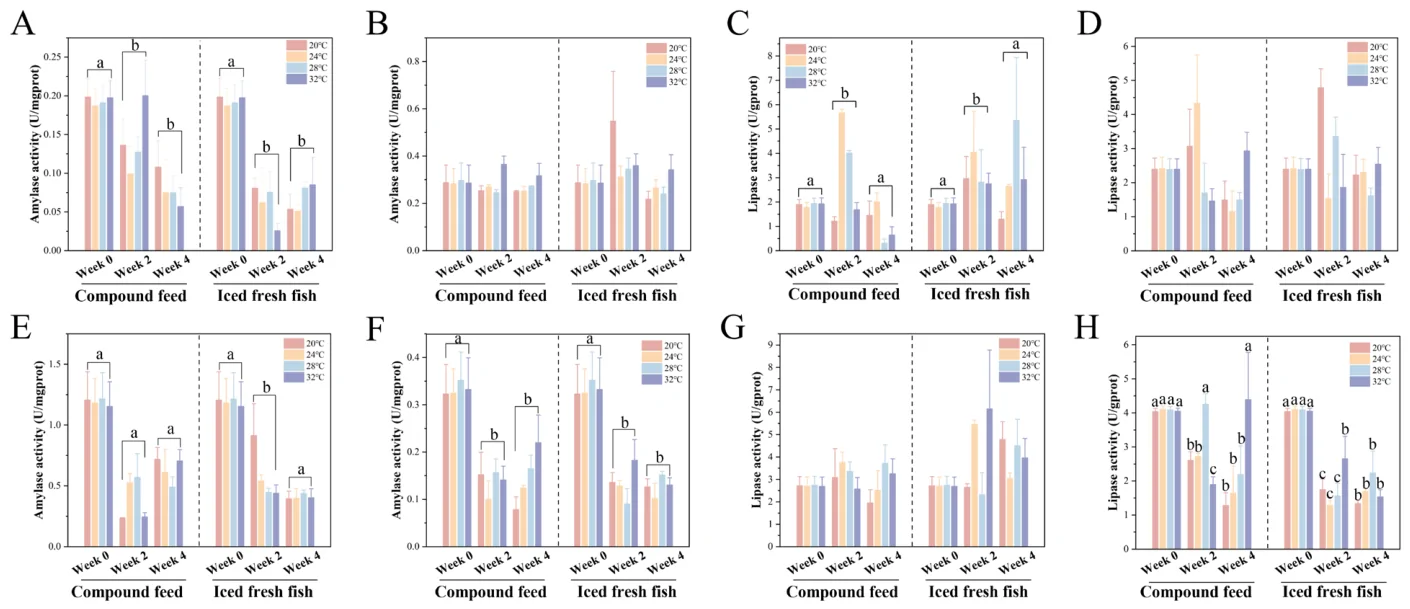
Effects of feeding regime and water temperature on digestive enzyme activities in juvenile and sub-adult Chinese mitten crab (*Eriocheir sinensis*) during the 4-week culture period. (**A**–**D**) represent hepatopancreatic amylase activity, intestinal amylase activity, hepatopancreatic lipase activity and intestinal lipase activity in juvenile crabs, respectively. (**E**–**H**) represent hepatopancreatic amylase activity, intestinal amylase activity, hepatopancreatic lipase activity and intestinal lipase activity in sub-adult crabs, respectively. Data are expressed as mean ± SE (*n* = 3 replicate tanks). Error bars represent standard error (SE). Different lowercase letters indicate significant differences among feeding regime–temperature combinations within the same time (Bonferroni-adjusted pairwise comparisons, *p* < 0.05). The effects of feeding regime, water temperature, time and their interactions were analyzed using a linear mixed-effects model (Table S7).

At week 0, amylase and lipase activities in the hepatopancreas and intestine did not differ significantly among feeding regime–temperature treatments in either juvenile or sub-adult *E. sinensis* (Figure 3).

In juvenile *E. sinensis*, hepatopancreatic amylase activity generally decreased during the culture period under both feeding regimes, whereas intestinal amylase activity remained relatively stable (Figure 3A,B). Lipase activities showed more variable temporal patterns among treatments. Hepatopancreatic lipase exhibited higher activity at week 2 under the compound-feed regime, while the iced-fresh-fish regime showed an increase toward week 4 at higher temperatures (Figure 3C). Intestinal lipase activity varied among treatments, with relatively higher values mainly observed at week 2 (Figure 3D).

In sub-adult *E. sinensis*, amylase activity generally declined over the culture period (Figure 3E,F). Hepatopancreatic amylase showed a significant feeding regime × time interaction and intestinal amylase was affected mainly by time. Hepatopancreatic lipase showed treatment-dependent temporal variation, with relatively higher activities observed under the iced-fresh-fish regime at elevated temperatures during the early culture period (Figure 3G). Intestinal lipase exhibited a distinct feeding regime- and temperature-dependent response, particularly under the iced-fresh-fish regime, where activity increased at 32 °C at week 2 and subsequently declined (Figure 3H).

The linear mixed-effects models indicated that, in juveniles, hepatopancreatic amylase was significantly affected by time and the feeding regime × time interaction, whereas no fixed effects were significant for intestinal amylase (Table S7). Hepatopancreatic lipase was significantly affected by water temperature and time, while intestinal lipase showed no significant fixed effects.

In sub-adults, time significantly affected all four digestive enzyme activities, including hepatopancreatic amylase, intestinal amylase, hepatopancreatic lipase, and intestinal lipase (Table S7). Hepatopancreatic amylase also showed a significant feeding regime × time interaction, whereas intestinal amylase was significantly affected only by time. Hepatopancreatic lipase showed a significant effect of time but no significant effects of feeding regime, water temperature, or their interactions. In contrast, intestinal lipase was significantly affected by feeding regime, time, feeding regime × time, and feeding regime × temperature × time interactions (Table S7).

### 3.4. Antioxidant Enzyme Activity

**Figure 4:**
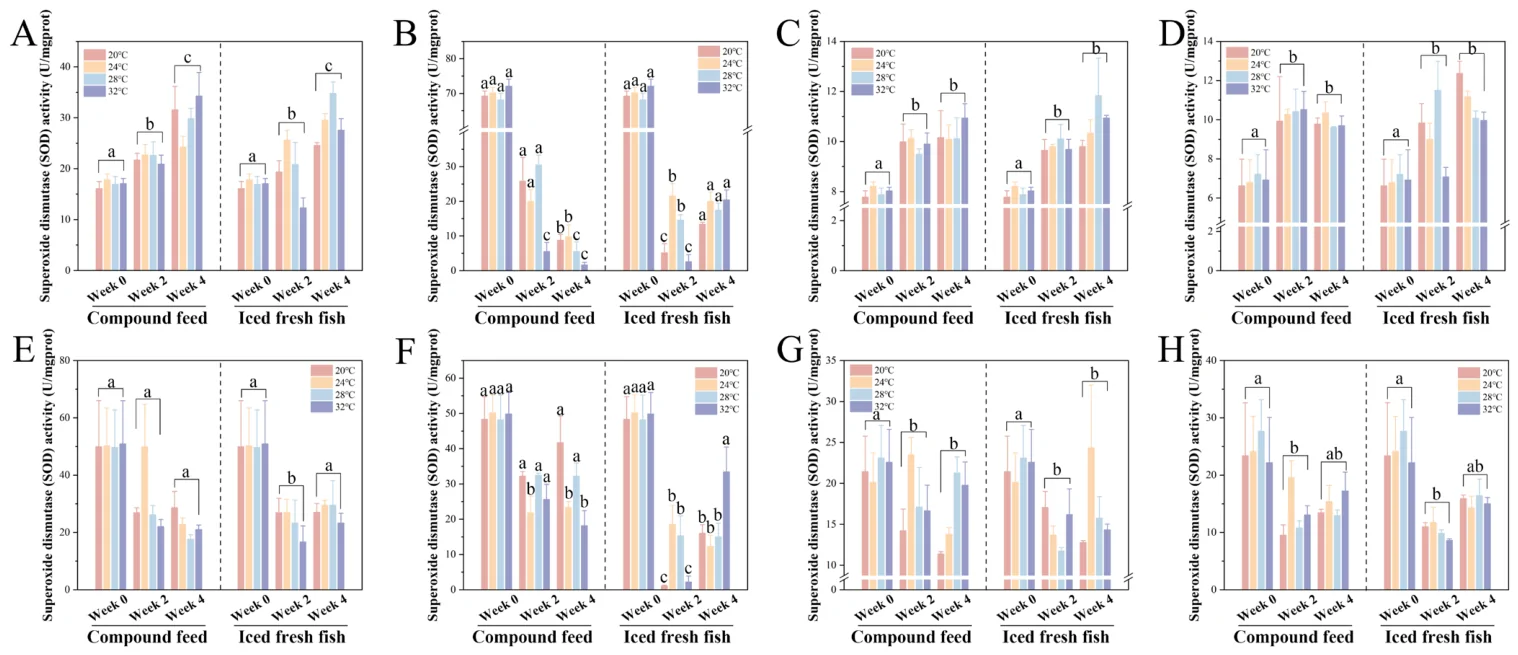
Effects of feeding regime and water temperature on superoxide dismutase (SOD) activity in different tissues of juvenile and sub-adult Chinese mitten crab (*Eriocheir sinensis*) during the 4-week culture period. (**A**–**D**) represent SOD activity in the hepatopancreas, intestine, gill and muscle of juvenile crabs, respectively. (**E**–**H**) represent SOD activity in the hepatopancreas, intestine, gill and muscle of sub-adult crabs, respectively. Data are expressed as mean ± SE (*n* = 3 replicate tanks). Error bars represent standard error (SE). Different lowercase letters indicate significant differences among feeding regime–temperature combinations within the same time (Bonferroni-adjusted pairwise comparisons, *p* < 0.05). The effects of feeding regime, water temperature, time and their interactions were analyzed using a linear mixed-effects model.

At week 0, superoxide dismutase (SOD) activity in the hepatopancreas, intestine, gill and muscle showed no marked differences among feeding regime–temperature treatments in either juvenile or sub-adult *E. sinensis* (Figure 4).

In juvenile *E. sinensis*, hepatopancreatic SOD activity generally increased from week 0 to week 4, with the magnitude of change varying among treatments (Figure 4A). Under the compound-feed regime, activity increased by 113.4% at 32 °C, while under the iced-fresh-fish regime, the largest increase occurred at 28 °C (116.6%). In contrast, intestinal SOD activity showed a marked decline during the culture period, with decreases exceeding 70.5% across feeding regimes (Figure 4B). Gill and muscle SOD activities generally increased over the culture period, with increases of approximately 52.2–86.6% observed in some treatments (Figure 4C,D).

In sub-adult *E. sinensis*, SOD responses differed among tissues and treatments (Figure 4E–H). Hepatopancreatic SOD activity decreased by 40.8–64.7% across all treatments (Figure 4E). Intestinal and muscle SOD activities also generally decreased over time, whereas gill SOD showed a more variable response, including an increase at 24 °C under the iced-fresh-fish regime (Figure 4F–H). Overall, the temporal changes in SOD activity differed between developmental stages and among tissues.

Linear mixed-effects model analysis showed that time significantly affected SOD activity in all four tissues of juvenile crabs (Table S7). Hepatopancreatic SOD also showed a significant feeding regime × temperature interaction. Intestinal SOD was significantly affected by water temperature, feeding regime × temperature interaction, feeding regime × time interaction, temperature × time interaction, and feeding regime × temperature × time interaction. In contrast, neither feeding regime nor temperature significantly affected gill or muscle SOD, although time had significant effects on both tissues.

In sub-adults, time significantly affected SOD activity in the hepatopancreas, intestine, gill, and muscle (Table S7). Hepatopancreatic SOD showed a significant feeding regime × time interaction. Intestinal SOD showed significant effects of feeding regime, time, feeding regime × temperature interaction, feeding regime × time interaction, and feeding regime × temperature × time interaction. No significant effects of feeding regime, temperature, or their interactions were detected for gill or muscle SOD, except for significant sampling-time effects.

### 3.5. Metabolic Enzyme Activity

#### 3.5.1. Lactate Dehydrogenase (LDH) Activity

**Figure 5:**
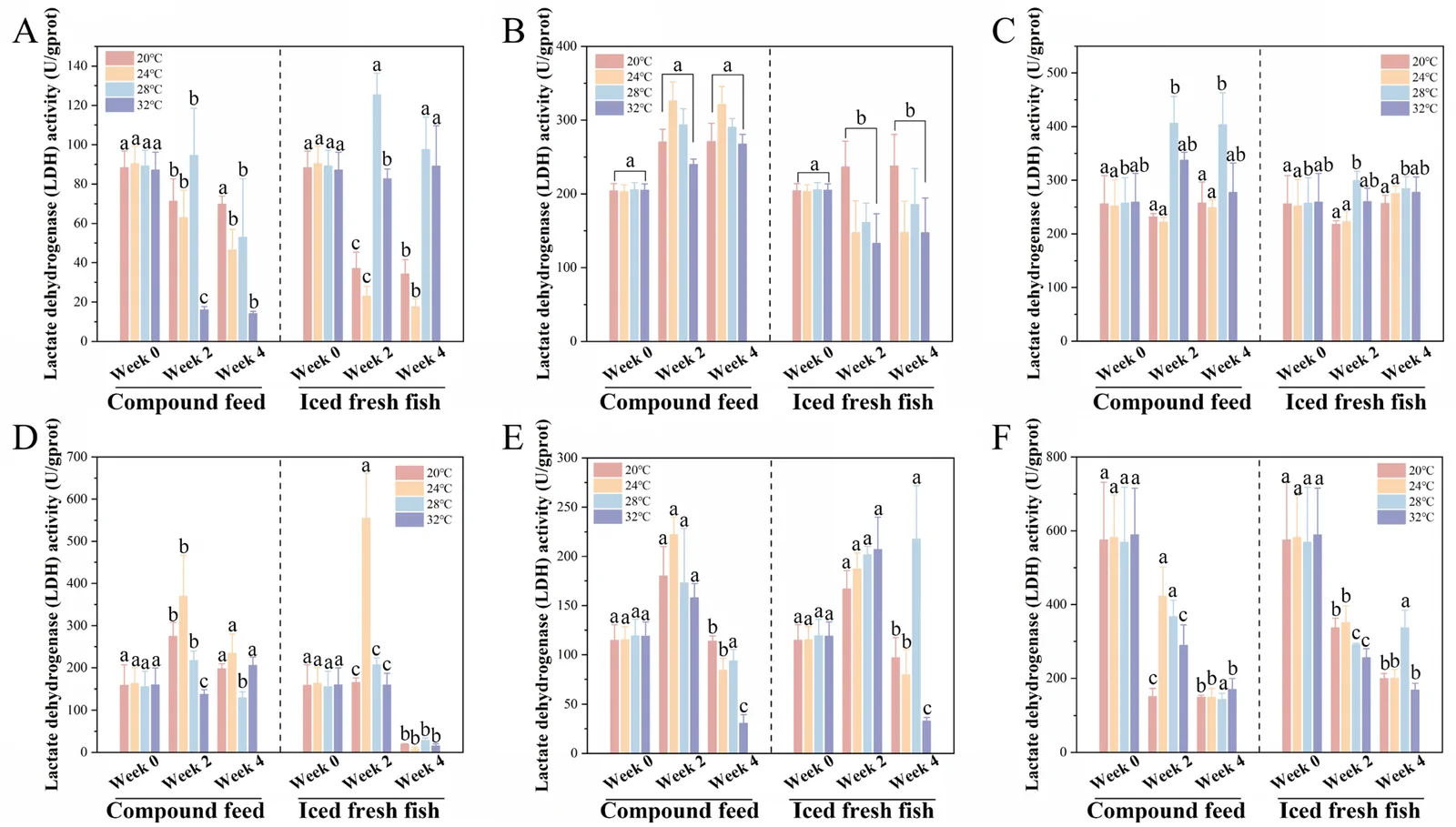
Effects of feeding regime and water temperature on lactate dehydrogenase (LDH) activity in different tissues of juvenile and sub-adult Chinese mitten crab (*Eriocheir sinensis*) during the 4-week culture period. (**A**–**C**) represent LDH activity in the hepatopancreas, gill and muscle of juvenile crabs, respectively. (**D**–**F**) represent LDH activity in the hepatopancreas, gill and muscle of sub-adult crabs, respectively. Data are expressed as mean ± SE (*n* = 3 replicate tanks). Error bars represent standard error (SE). Different lowercase letters indicate significant differences among feeding regime–temperature combinations within the same time (Bonferroni-adjusted pairwise comparisons, *p* < 0.05). The effects of feeding regime, water temperature, time and their interactions were analyzed using a linear mixed-effects model.

At week 0, lactate dehydrogenase (LDH) activity in the hepatopancreas, gill and muscle showed no significant differences among feeding regime–temperature treatments in either juvenile or sub-adult *E. sinensis* (Figure 5).

In juvenile *E. sinensis*, hepatopancreatic LDH activity showed distinct temporal patterns between feeding regimes (Figure 5A). Under the compound-feed regime, LDH activity generally over time, particularly at 32 °C at week 4. Under the iced-fresh-fish regime, hepatopancreatic LDH activity increased at 28 °C at week 2 and subsequently decreased. Gill LDH activity generally showed higher values under the compound-feed regime, particularly at the later times (Figure 5B). Muscle LDH activity remained relatively stable across temperatures and times under the iced-fresh-fish regime, whereas the compound-feed regime showed relatively high activity at 28 °C (Figure 5C).

In sub-adult *E. sinensis*, hepatopancreatic LDH activity under the compound-feed regime generally decreased over time, particularly at 32 °C (Figure 5D). Under the iced-fresh-fish regime, hepatopancreatic LDH activity showed higher values at 28 °C during later sampling periods. Gill LDH activity varied over time, with relatively higher values observed under the compound-feed regime at some sampling points (Figure 5E). Muscle LDH activity under the iced-fresh-fish regime varied relatively little among treatments, whereas the compound-feed regime showed relatively high activity (Figure 5F).

Linear mixed-effects model analysis revealed tissue- and stage-specific effects on LDH activity (Table S7). In juveniles, hepatopancreatic LDH activity was significantly affected by water temperature and time, with significant feeding regime × temperature, temperature × time, and feeding regime × temperature × time interactions. The main effects of the feeding regime and the feeding regime × time interaction were not significant. Gill LDH activity was significantly affected by feeding regime and time, whereas interaction effects were limited. Muscle LDH activity was significantly affected by water temperature.

In sub-adults, hepatopancreatic LDH activity was significantly affected by feeding regime, water temperature, and time, with significant interactions involving time (Table S7). Gill LDH activity was significantly affected by time and the temperature × time interaction, whereas feeding regime, temperature, and the other interaction terms were not significant. Muscle LDH activity was significantly affected by time and showed a significant feeding regime × temperature × time interaction.

#### 3.5.2. ATPase Activity

**Figure 6:**
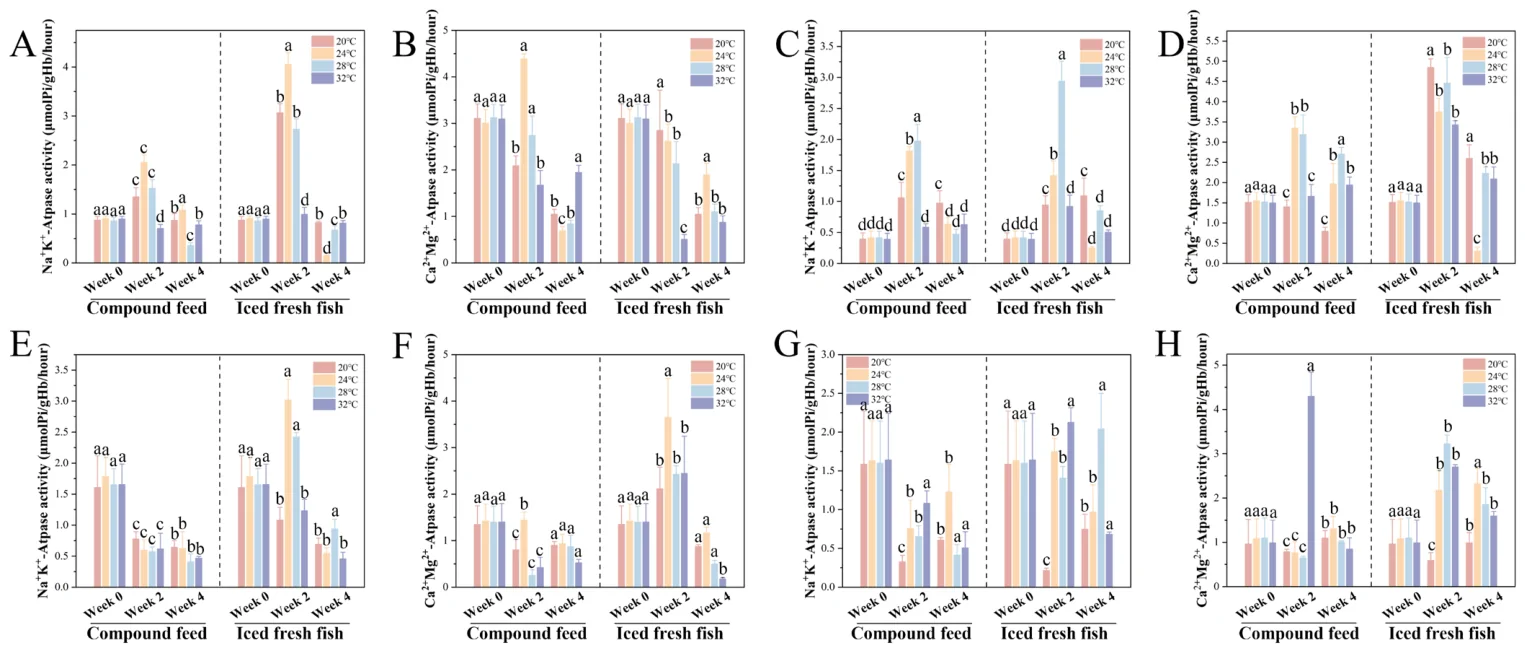
Effects of feeding regime and water temperature on ATPase activities in juvenile and sub-adult Chinese mitten crab (*Eriocheir sinensis*) during the 4-week culture period. (**A**–**D**) represent hepatopancreatic Na^+^/K^+^-ATPase activity, hepatopancreatic Ca^2+^/Mg^2+^-ATPase activity, muscle Na^+^/K^+^-ATPase activity and muscle Ca^2+^/Mg^2+^-ATPase activity in juvenile crabs, respectively. (**E**–**H**) represent hepatopancreatic Na^+^/K^+^-ATPase activity, hepatopancreatic Ca^2+^/Mg^2+^-ATPase activity, muscle Na^+^/K^+^-ATPase activity and muscle Ca^2+^/Mg^2+^-ATPase activity in sub-adult crabs, respectively. Data are expressed as mean ± SE (*n* = 3 replicate tanks). Error bars represent standard error (SE). Different lowercase letters indicate significant differences among feeding regime–temperature combinations within the same time period (Bonferroni-adjusted pairwise comparisons, *p* < 0.05). The effects of feeding regime, water temperature, time and their interactions were analyzed using a linear mixed-effects model.

At week 0, Na^+^/K^+^-ATPase and Ca^2+^/Mg^2+^-ATPase activities in the hepatopancreas and muscle showed no marked differences among feeding regime–temperature treatments in either juvenile or sub-adult *E. sinensis* (Figure 6).

In juvenile *E. sinensis*, hepatopancreatic Na^+^/K^+^-ATPase activity varied among feeding regime, temperature, and sampling-time combinations (Figure 6A). Hepatopancreatic Ca^2+^/Mg^2+^-ATPase activity showed different patterns, with a general decline observed under the iced-fresh-fish regime (Figure 6B). In muscle, Na^+^/K^+^-ATPase activity was relatively high in the iced-fresh-fish regime and Ca^2+^/Mg^2+^-ATPase activity showed temporal variations among treatments (Figure 6C,D).

In sub-adult *E. sinensis*, hepatopancreatic Na^+^/K^+^-ATPase and Ca^2+^/Mg^2+^-ATPase activities showed pronounced temporal variations between feeding regimes (Figure 6E,F). Under the compound-feed regime, both ATPase activities generally decreased during the culture period. In contrast, the iced-fresh-fish regime showed higher ATPase activities during the early culture period followed by subsequent declines. Muscle Na^+^/K^+^-ATPase activity showed a relatively high value in the iced-fresh-fish regime at 32 °C at week 2, whereas the compound-feed regime showed comparatively lower activity under several treatment combinations (Figure 6G). Muscle Ca^2+^/Mg^2+^-ATPase activity showed marked temporal and treatment-dependent variation, with relatively high activity in the compound-feed regime at 32 °C at week 2 and in the iced-fresh-fish regime at 28 °C (Figure 6H).

The linear mixed-effects models revealed that hepatopancreatic Na^+^/K^+^-ATPase activity in juveniles was significantly affected by feeding regime, water temperature, time, and their interactions (Table S7). Juvenile muscle Na^+^/K^+^-ATPase activity was significantly affected by water temperature, time, and feeding regime × temperature and temperature × time interactions, whereas the three-way interaction was not significant. Hepatopancreatic Ca^2+^/Mg^2+^-ATPase activity was significantly affected by temperature and time, as well as by the temperature × time and feeding regime × temperature × time interactions. Muscle Ca^2+^/Mg^2+^-ATPase activity showed the significant effects of feeding regime, temperature, time, and all two- and three-way interactions (Table S7).

In sub-adults, hepatopancreatic Na^+^/K^+^-ATPase activity was significantly affected by feeding regime, time, feeding regime × time, temperature × time, and the three-way interaction, whereas the overall temperature effect and feeding regime × temperature interaction were not significant. Muscle Na^+^/K^+^-ATPase activity showed a significant effect of time and temperature × sampling-time interaction, while the three-way interaction was not significant. Hepatopancreatic Ca^2+^/Mg^2+^-ATPase activity was significantly affected by feeding regime, time, and feeding regime × time interaction, whereas temperature and the remaining interactions were not significant. In contrast, muscle Ca^2+^/Mg^2+^-ATPase activity was significantly affected by feeding regime, temperature, time, feeding regime × temperature, temperature × time, and the three-way interaction (Table S7).

### 3.6. Immunoenzyme Activity

**Figure 7:**
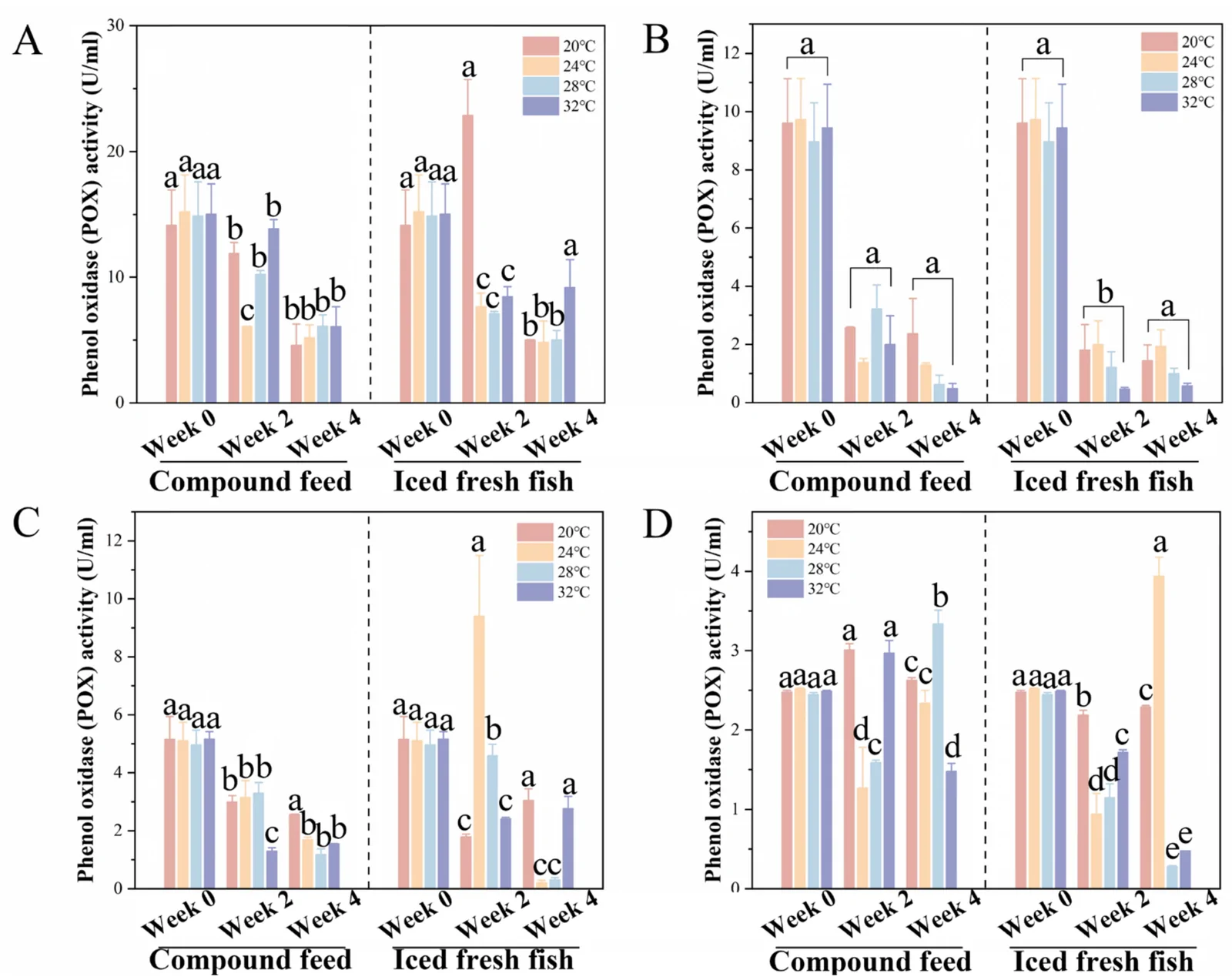
Effects of feeding regime and water temperature on phenoloxidase (POX) activity in juvenile and sub-adult Chinese mitten crab (*Eriocheir sinensis*) during the 4-week culture period. (**A**,**B**) represent hepatopancreatic and gill POX activities in juvenile crabs, respectively. (**C**,**D**) represent hepatopancreatic and gill POX activities in sub-adult crabs, respectively. Data are expressed as mean ± SE (*n* = 3 replicate tanks). Error bars represent standard error (SE). Different lowercase letters indicate significant differences among feeding regime–temperature combinations within the same time (Bonferroni-adjusted pairwise comparisons, *p* < 0.05). The effects of feeding regime, water temperature, time and their interactions were analyzed using a linear mixed-effects model.

At week 0, phenoloxidase (POX) activity in the hepatopancreas and gill showed no significant differences among feeding regime–temperature treatments in either juvenile or sub-adult *E. sinensis* (Figure 7).

In juvenile *E. sinensis*, hepatopancreatic POX activity generally decreased over time under the compound-feed regime (Figure 7A). In the iced-fresh-fish regime, hepatopancreatic POX activity showed a transient increase during the early culture period followed by a decline. Gill POX activity generally decreased during the culture period across most treatments (Figure 7B).

In sub-adult *E. sinensis*, hepatopancreatic POX activity also showed pronounced temporal variation (Figure 7C). Under the compound-feed regime, activity generally decreased during the culture period. In contrast, the iced-fresh-fish regime showed a marked increase in hepatopancreatic POX activity during the early culture period followed by a decline at later sampling times. Gill POX activity also varied among feeding regime–temperature–time combinations, with activity increasing from week 2 to week 4 at 28 °C under the compound-feed regime, whereas it decreased over the same period at 28 and 32 °C under the iced-fresh-fish regime (Figure 7D).

The linear mixed-effects models showed that juvenile hepatopancreatic POX activity was significantly affected by water temperature and time and their interactions with feeding regime (Table S7). Juvenile gill POX activity was significantly affected by time and the feeding regime × time interaction.

In sub-adult *E. sinensis*, hepatopancreatic POX activity was significantly affected by feeding regime, water temperature, and time, with all three being main effects. Significant interactions were also detected for feeding regime × temperature, feeding regime × time, temperature × time and feeding regime × temperature × time (Table S7). Gill POX activity showed the significant effects of feeding regime, water temperature, and time, as well as the significant interactions of the two- and three-way interaction.

### 3.7. Histological Observations

#### 3.7.1. Intestinal Histology

**Figure 8:**
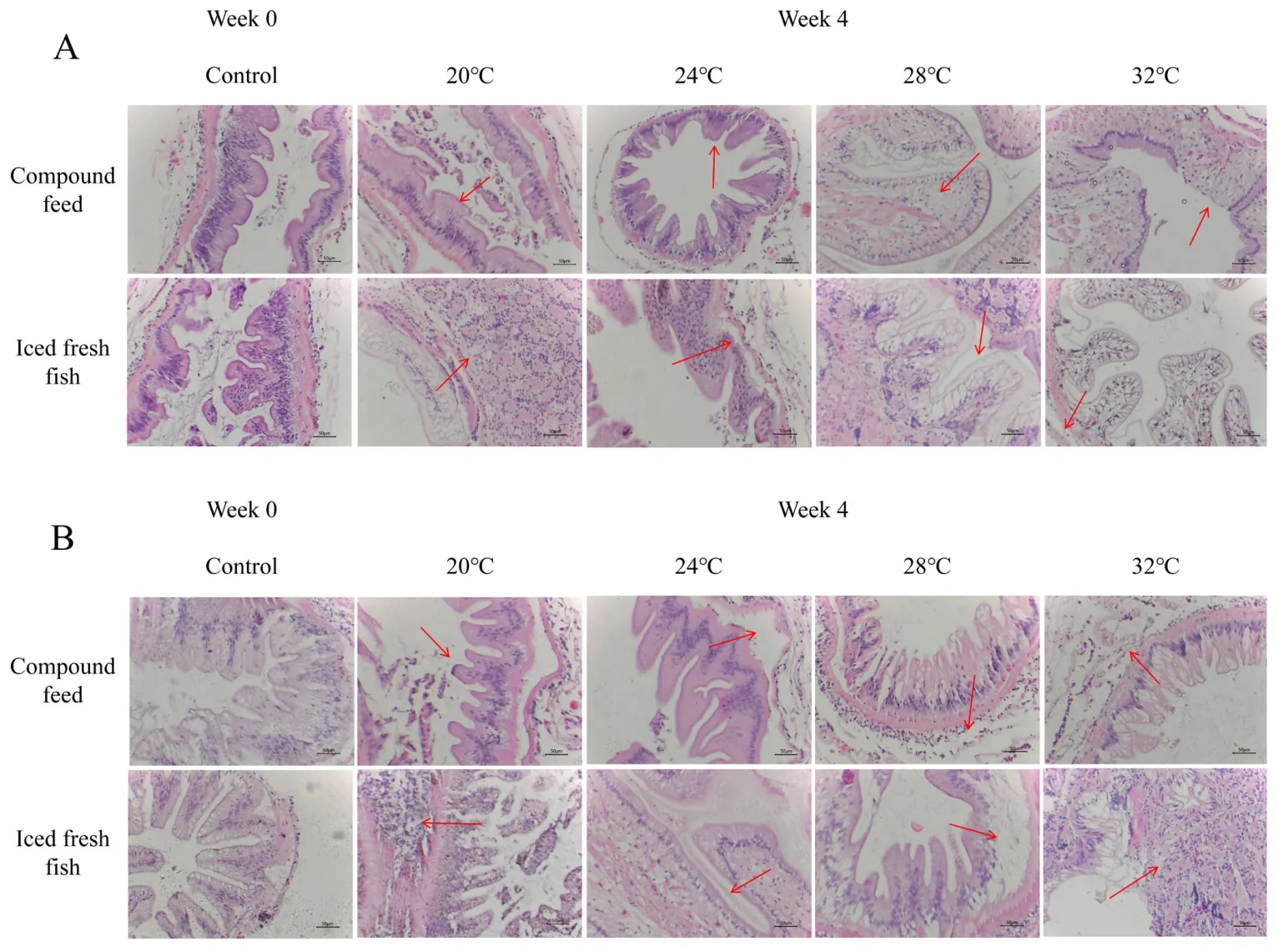
Effects of feeding regime and water temperature on the intestinal morphology of juvenile and sub-adult Chinese mitten crab (*Eriocheir sinensis*) after the 4-week culture period. (**A**,**B**) represent representative hematoxylin and eosin-stained intestinal sections of juvenile and sub-adult crabs, respectively. One representative section was selected from each treatment group for image presentation. Histological alterations included epithelial thinning, mucosal-fold blunting, epithelial separation, hemocyte aggregation and fragmented tissue appearance. Arrows indicate representative histological alterations, including epithelial disruption, hemocyte infiltration and mucosal fragmentation. Scale bars = 50 μm. Histological observations were descriptive and were not subjected to morphometric or statistical severity analysis.

Histological changes in the intestine of juvenile and sub-adult *E. sinensis* were examined using hematoxylin and eosin staining at weeks 0 and 4. Compared with the post-acclimation baseline, intestinal sections at week 4 showed treatment-associated alterations in epithelial structure, mucosal folds, and luminal morphology (Figure 8).

In juvenile *E. sinensis*, the compound-feed group showed relatively limited morphological alterations at 20–24 °C, including reduced definition of mucosal folds and epithelial thinning. At 28–32 °C, epithelial disruption and cell shedding became more evident, and the mucosal folds were less distinct at 32 °C, resulting in a relatively flattened intestinal wall. In the iced-fresh-fish group, hemocyte aggregation and partial narrowing of the intestinal lumen were observed at 20 °C, whereas epithelial swelling and separation of the epithelial layer were apparent at 24 °C. At 28–32 °C, intestinal sections showed more evident mucosal fragmentation and epithelial structural alteration, with hypertrophic changes in the basal epithelial region observed at 32 °C.

In sub-adult *E. sinensis*, the compound-feed group showed reduced definition of mucosal folds and epithelial thinning at 20–24 °C. At 28–32 °C, epithelial cell shedding became more evident. In the iced-fresh-fish group, increased hemocyte presence was observed at 20 °C, while separation of the epithelial layer was apparent at 24 °C. At 28–32 °C, intestinal sections showed epithelial disruption, discontinuous tissue organization, and loss of regular mucosal organization, with greater structural alterations observed at 32 °C.

These observations are descriptive and were not subjected to morphometric measurements or statistical severity scoring.

#### 3.7.2. Gill Histology

**Figure 9:**
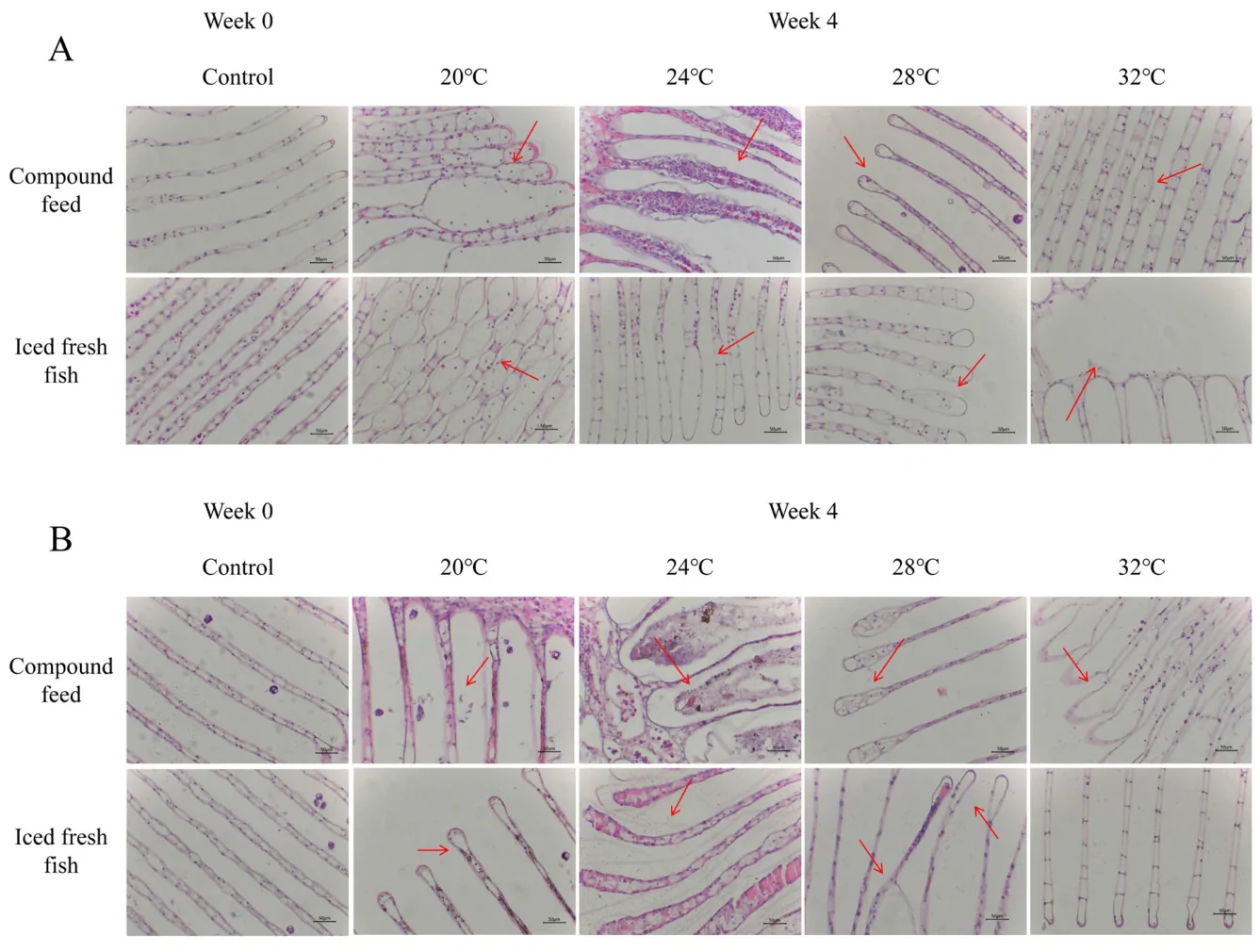
Effects of feeding regime and water temperature on the gill morphology of juvenile and sub-adult Chinese mitten crab (*Eriocheir sinensis*) after the 4-week culture period. (**A**,**B**) represent representative hematoxylin and eosin-stained gill sections of juvenile and sub-adult crabs, respectively. One representative section was selected from each treatment group for image presentation. Histological alterations included filament swelling, epithelial separation, lamellar fusion, hemocyte accumulation and altered pillar-cell organization. Arrows indicate representative histological alterations. Scale bars = 50 μm. Histological observations were descriptive and were not subjected to morphometric or statistical severity analysis.

Histological changes in the gill tissues of juvenile and sub-adult *E. sinensis* were examined using hematoxylin and eosin staining at weeks 0 and 4. Compared with the post-acclimation baseline, gill sections at week 4 showed treatment-associated alterations, including filament swelling, epithelial separation, lamellar fusion, and hemocyte accumulation (Figure 9).

In juvenile *E. sinensis*, the compound-feed group at 20 °C showed swelling at the distal ends of the gill filaments, together with increased intercellular spaces between epithelial cells and pillar-cell regions. In the iced-fresh-fish group at 20 °C, multiple localized swellings were observed along the gill filaments. At 24 °C, the compound-feed group showed apparent thickening near the base of the gill lamellae and partial fusion of adjacent filaments, accompanied by a reduction in interlamellar space. In contrast, the iced-fresh-fish group at 24 °C retained a relatively regular lamellar structure, although swelling at the filament tips was observed. At 28–32 °C, gill filaments in the compound-feed group maintained relatively recognizable filament organization, with scattered filament enlargement, whereas enlargement of the gill axis was observed in the iced-fresh-fish group at 32 °C.

In sub-adult *E. sinensis*, gill morphology also varied among feeding regime–temperature combinations. At 20 °C, hemocyte accumulation was observed in the central region of the gill axis in the compound-feed group, whereas swelling at the distal ends of the gill filaments was observed in the iced-fresh-fish group. At 24 °C, dark-stained cellular aggregates, likely corresponding to hemocyte accumulation, were observed in the compound-feed group. In contrast, the iced-fresh-fish group showed epithelial separation, discontinuous tissue organization, and disorganization of the pillar-cell region. At 28–32 °C, the gill lamellae in the iced-fresh-fish group remained as relatively clear lamellar structures, with filament swelling being the main alteration at 28 °C. The compound-feed group showed enlargement of the filament tips at both 28 and 32 °C.

As with the intestinal observations, these findings are descriptive and were not statistically quantified.

## 4. Discussion

### 4.1. Stage-Specific Growth and Survival Responses to Feeding Regime and Water Temperature

Growth performance is an integrated response to feed supply, nutrient utilization, energy allocation, and physiological adjustment under a given culture condition. In the present study, growth responses were strongly influenced by feeding regime–temperature combinations, indicating that the effects of practical feeding regimes depended on thermal conditions rather than acting independently. This finding is consistent with previous studies indicating that growth and developmental processes in *E. sinensis* are strongly influenced by both nutritional conditions and water temperature [13,24,25]. Although these studies focused mainly on juvenile crabs and did not directly compare the practical feeding regimes used here, they provide relevant evidence for temperature- and stage-dependent growth responses.

Within the compound-feed regime, juvenile mean body-weight gain and specific growth rate were numerically greatest at 28 °C. In contrast, juveniles receiving iced fresh fish showed a different temperature-dependent pattern, with relative growth generally decreasing as temperature increased. Previous studies have shown that water temperature strongly affects feeding, molting and growth in juvenile *E. sinensis*, with performance improving within an appropriate temperature range but declining when temperature becomes stressful [13,26]. The relatively high growth observed under the compound-feed regime at 28 °C may therefore reflect a combination of temperature-dependent physiological activity and the characteristics of the formulated feed, particularly its higher protein density and greater physical stability in water. However, because compound feed and iced fresh fish differed substantially in moisture content, nutrient density, and physical properties, the as-fed feeding strategy did not provide equivalent dry-matter or nutrient intake between treatments. Therefore, the present results should be interpreted as responses to different practical feeding regimes rather than evidence of the intrinsic superiority of one feed type over the other.

The growth response of sub-adult crabs differed from that of juveniles. Under the compound-feed regime, the highest body-weight gain and SGR were observed at 24 °C, whereas the iced-fresh-fish regime resulted in relatively high growth at 20 °C. Thus, the temperature-associated growth pattern depended on both feeding regime and developmental stage. Similar temperature-dependent growth responses have also been reported in other cultured crabs, including *Scylla paramamosain* [27,28]. Differences between developmental stages may be related to variations in body size, nutritional requirements, energy allocation, and physiological capacity. Nevertheless, these mechanisms were not directly measured in the present study and should therefore be considered possible explanations rather than demonstrated mechanisms. In addition to these biological considerations, potential variations in initial body size among individuals should also be taken into account when interpreting growth responses. Although relative growth indices (WGR, SGR, and relative carapace increments) were used to facilitate comparison among treatments with different initial body sizes, these indices may not have completely removed the influence of baseline variation among individuals. Therefore, growth responses should be interpreted together with the initial size information provided in the Supplementary Materials.

The low pooled survival observed in sub-adults at 32 °C warrants particular caution when interpreting growth and tissue responses under this experimental condition. This difference may reflect stage-dependent metabolic demand, energy allocation, or physiological capacity during prolonged high-temperature exposure. As these mechanisms were not directly measured, the observed stage difference should be regarded as a possible stage-dependent response. Survival data were pooled at the treatment level and were therefore interpreted descriptively. Furthermore, physiological and histological measurements were obtained only from surviving crabs. Therefore, responses observed under 32 °C may partly reflect survivor selection and should not be assumed to represent the response of the entire initial population.

### 4.2. Apparent Feeding Rate and Interpretation of Feed Intake

The higher ADFR observed in the iced-fresh-fish groups should not be interpreted directly as greater voluntary feed intake. Importantly, feed supplied was recorded on an as-fed (wet-weight) basis, whereas recovered residual feed was dried to a constant weight before weighing. In the present study, ADFR was calculated from the difference between the amount of feed supplied and the amount of residual feed recovered from each tank. Therefore, the measured value represents apparent feed disappearance and may include both feed consumed by the crabs and feed lost from the feeding system. This distinction is particularly important for iced fresh fish because raw fish tissue is considerably less physically stable in water than formulated feed. After immersion, fresh fish can soften, fragment, and release soluble or fine particulate components into the surrounding water. In addition, the tearing and manipulation of soft fish tissue during feeding may further increase the production of small particles that are difficult to recover completely. Consequently, some feed components may disappear from the feeding area without being ingested, potentially resulting in an overestimation of actual feed consumption.

The difference in physical and nutritional characteristics between the two feeding regimes also limits direct comparison of ADFR on an as-fed basis. Compound feed and iced fresh fish differ substantially in moisture content and nutrient density; therefore, the disappearance of the same mass of wet feed does not necessarily represent an equivalent intake of dry matter, protein, lipids, or energy. Instead, ADFR should be regarded as an operational indicator of feed disappearance under the specific feeding and recovery conditions used in this study.

Future studies incorporating dry-matter intake, batch-specific nutrient composition, feed conversion efficiency, and quantitative measurements of feed leaching would provide a more direct assessment of the relationship between feeding behavior, feed utilization, and growth in *E. sinensis*.

### 4.3. Enzyme Responses Indicate Tissue- and Stage-Specific Physiological Adjustment

Digestive enzymes provide useful information on the capacity of animals to process dietary nutrients. In crustaceans, amylase and lipase activities are affected by developmental stage, diet composition and environmental conditions [29,30,31]. In the present study, enzyme activity generally showed stronger temporal and tissue-specific variation than consistent main effects of either feeding regime or temperature. This pattern indicates that the physiological responses of *E. sinensis* were dynamic and depended on the interaction between feeding regime, thermal environment, developmental stage, and tissue function.

Previous work has shown that dietary protein level and protein source can affect digestive enzyme activities in *E. sinensis* and other decapod crustaceans [4,32]. However, because digestive enzyme activity was not accompanied by direct measurements of nutrient digestibility or substrate utilization, a decrease in enzyme activity could not by itself be interpreted as reduced digestive performance.

Lipase activity showed more variable responses than amylase, particularly in the hepatopancreas. These patterns suggest that lipid digestion-related enzymatic regulation was sensitive to both feeding regime and temperature. Previous research in decapod crustaceans has shown that temperature and diet can modify digestive enzyme profiles and apparent nutrient utilization [31,33]. Nevertheless, the actual lipid composition and digestibility were not directly measured in this study. Therefore, the underlying causes of these lipase responses require further confirmation.

The antioxidant response also differed among tissues and developmental stages. An increase in antioxidant enzyme activity may indicate an enhanced antioxidant response, but it does not by itself demonstrate that oxidative stress was successfully controlled or that the animals were physiologically better adapted to the treatment. Conversely, lower SOD activity cannot necessarily be interpreted as antioxidant impairment. Oxidative stress involves a balance between reactive oxygen species production and antioxidant capacity, and the present study did not directly measure reactive oxygen species, malondialdehyde, or other oxidative damage indicators. Therefore, the SOD results provide evidence of altered antioxidant regulation but do not establish the presence, magnitude, or direction of oxidative damage. Nutritional conditions may also have contributed to the observed antioxidant differences. Recent studies have shown that dietary regulation, including methionine, yeast culture, carotenoids and chitinase supplementation, can influence antioxidant and immune responses in Chinese mitten crab under normal or stressful conditions [12,16,34,35]. These findings support the idea that nutritional strategy can modify stress responses, but the specific active components responsible for the responses observed in the present study were not determined.

LDH is commonly used as an indicator of metabolic enzyme activity associated with lactate metabolism and cellular energy adjustment. The hepatopancreas is a major organ for nutrient processing and intermediary metabolism, whereas the gill is directly exposed to the surrounding water and participates in respiration and ion regulation. Studies on *E. sinensis* have shown that gill and hepatopancreas tissues are important organs for stress and thermal adaptation [19,36]. Therefore, the LDH responses observed here suggest that metabolic adjustment differed between central metabolic and respiratory tissues. However, LDH activity should also be interpreted as an indicator of metabolic adjustment rather than as direct evidence of anaerobic metabolism or metabolic stress. Although LDH is commonly associated with lactate metabolism and anaerobic energy production, changes in tissue LDH activity may also reflect altered lactate turnover, glycolytic flux, energy-substrate utilization, or tissue-specific metabolic adjustment.

ATPase activity revealed another layer of physiological regulation. Na^+^/K^+^-ATPase and Ca^2+^/Mg^2+^-ATPase activities varied substantially among tissues, developmental stages, feeding regimes, temperatures, and times. These results indicate that ion-transport-related enzyme activity was highly dependent on the combination of environmental and nutritional conditions rather than being regulated in a uniform direction. The observed ATPase responses are consistent with the involvement of energy-dependent ion transport in cellular regulation. In crustaceans, ion transport is closely associated with environmental regulation and tissue function [21,37]. However, ATPase activity was measured only in the hepatopancreas and muscle, and gill ATPase activity was not assessed. Therefore, these results should be interpreted as tissue-specific changes in ion-transport-related enzyme activity rather than direct evidence of gill-specific or whole-animal osmoregulatory responses. The absence of direct measurements of hemolymph ions, membrane lipid composition, transporter abundance, or ion-regulatory gene expression also limits mechanistic interpretation. The results are more appropriately regarded as evidence that energy-dependent ion-transport processes were modulated under different feeding regime–temperature conditions.

Phenoloxidase (POX) activity represented another aspect of physiological regulation related to innate immunity. The prophenoloxidase system is an important component of crustacean innate immune defense and is involved in melanization and related immune processes [26,38]. The POX responses varied with tissue and developmental stage. Transient increases in POX activity under specific feeding regime–temperature combinations may indicate changes in innate immune activity, but POX activity alone cannot be used to establish an overall enhancement or suppression of immune status. The prophenoloxidase system is only one component of crustacean immunity, and its activity may be influenced by multiple physiological and environmental factors [26,38]. In the present study, hemocyte abundance, proPO-related gene expression, antimicrobial activity, and pathogen load were not measured. Therefore, the POX results should be interpreted as evidence of altered immune-related enzyme activity rather than direct evidence of improved or impaired disease resistance.

Finally, the physiological measurements should be interpreted in the context of the temporal design and survival pattern of the experiment. Week 0 represented a post-acclimation baseline rather than an untreated pre-experimental state, and subsequent changes therefore reflect responses during the 28-day culture period after acclimation to the assigned conditions. As discussed in Section 4.1, these enzyme measurements describe sampled survivors and do not establish population-level thermal tolerance. Future studies combining enzyme activities with oxidative damage markers, metabolic rate, hemolymph ion concentrations, immune indicators, and molecular markers would help clarify the mechanisms underlying the tissue- and stage-specific responses observed in *E. sinensis*.

### 4.4. Intestinal and Gill Histology as Indicators of Tissue Condition

Histological observations provided complementary evidence of tissue-level responses to different feeding regimes and water temperatures. The intestine is responsible for digestion, nutrient absorption and barrier function, and its structural condition can provide useful information on intestinal status. In this study, intestinal sections showed changes in epithelial organization, mucosal folds, epithelial separation, hemocyte accumulation, and tissue integrity under several feeding regime–temperature combinations.

The intestinal alterations observed here are consistent with previous reports showing that temperature and dietary conditions can influence intestinal health, microbial composition, and immune-related responses in *E. sinensis* and other aquatic animals [17,18,39]. However, the histological assessment was qualitative and was based on representative tissue sections rather than quantitative morphometric measurements or standardized lesion scores. Therefore, the observed histological changes should be interpreted as descriptive evidence of altered intestinal condition rather than as statistically demonstrated differences in tissue damage severity.

The gill is another key tissue for evaluating crab health because it is involved in respiration, ion regulation, nitrogenous waste excretion and local immune defense. In the present study, gill sections showed filament swelling, epithelial separation, lamellar fusion, hemocyte accumulation and disruption of pillar-cell regions under different feeding regime–temperature treatments. Previous studies have shown that the gill of *E. sinensis* is highly responsive to environmental stress and thermal adaptation, with changes involving ion transport, antioxidant response and cellular stress pathways [19,21].

The histological responses of the gill should nevertheless be interpreted cautiously because structural changes do not by themselves demonstrate functional impairment. Although epithelial separation, lamellar fusion, filament swelling, and changes in pillar-cell organization may be associated with altered gill function, the present study did not directly measure hemolymph osmolality, ion concentrations, oxygen consumption, or gill transporter expression. Therefore, these observations cannot be used as direct evidence of osmoregulatory failure or respiratory dysfunction. Instead, they provide qualitative evidence that gill tissue condition varied under different feeding and temperature conditions.

These tissue-level observations provide additional context for interpreting the growth and enzyme responses, but they do not establish specific mechanisms of tissue injury or physiological dysfunction. Quantitative histomorphometry, standardized lesion scoring, intestinal microbiota analysis, barrier-function markers, hemolymph ion measurements, and gill transporter expression would be useful in future studies to determine the mechanisms underlying the observed tissue-level responses.

### 4.5. Implications and Future Experimental Considerations

From a practical aquaculture perspective, the present results indicate that feeding regime and water temperature should be considered jointly rather than independently. Under the conditions tested, compound feed showed relatively favorable growth responses at 28 °C in juveniles and 24–28 °C in sub-adults, whereas iced fresh fish exhibited variable responses across temperatures. However, these findings should be considered preliminary because the experiment lasted only 28 days under controlled conditions and the two feeds differed in moisture and nutrient density. Longer-term studies incorporating growth, molting, feed conversion, and survival, together with improved assessment of feed disappearance from fresh diets, are needed to further evaluate practical feeding strategies.

## 5. Conclusions

This study demonstrates that feeding regime and water temperature interact significantly to govern growth performance in both juvenile and sub-adult *E. sinensis*. Under the present experimental conditions, the highest growth among surviving individuals occurred under the compound-feed regime at 28 °C for juveniles and at 24 °C for sub-adult body-weight gain, though both performance outcomes must be interpreted strictly in conjunction with survival rates. Although apparent feed disappearance was consistently higher in the iced-fresh-fish regime, this did not equate to greater true feed intake or superior growth. Low pooled survival at 32 °C, particularly in sub-adults, limits interpretation of the associated tissue measurements. These findings describe sampled survivors, may be influenced by survivor selection, and do not establish population-level thermal tolerance or the physiological causes of mortality. Similarly, structural alterations observed in the intestine and gill provide qualitative, descriptive evidence rather than quantitative indices of tissue damage. To resolve these underlying dynamics, future studies should evaluate tank-level replicated survival, precise molting records, batch-specific proximate nutrient and fatty acid profiles, and feed leaching corrections. Finally, coupling these parameters with water quality tracking over longer experimental durations will be essential to establish optimized stage- and temperature-dependent feeding strategies.

## Figures and Tables

**Table 1:** Nutritional specifications of the commercial diets and estimated proximate composition of iced fresh fish (on an as-fed basis and dry-matter basis).

Component	Juvenile Pellet	Sub-Adult Pellet	Iced Fresh Fish
Moisture (As-fed, %)	≤12.0	≤12.0	80.17 ± 0.44
Crude Protein (DM, %)	≥42.0	≥36.0	62.50 ± 2.18
Crude Lipid (DM, %)	≥5.0	≥4.5	20.22 ± 1.10
Crude Ash (DM, %)	≤18.0	≤18.0	13.85 ± 0.91

Note: Values for commercial compound feeds are based on manufacturer-guaranteed specifications. Values for iced fresh fish represent compiled standard values from the literature reference [23] for the specific fish species.

**Table 2:** Pooled survival rates and quantities of juvenile and sub-adult Chinese mitten crab (*Eriocheir sinensis*) after the 28-day culture trial.

		Juvenile *E. sinensis*	Sub-Adult *E. sinensis*
Compound Feed	20 °C	75.56% (68/90)	82.22% (37/45)
	24 °C	65.56% (59/90)	84.44% (38/45)
	28 °C	58.89% (53/90)	84.44% (38/45)
	32 °C	58.89% (53/90)	37.78% (17/45)
Iced Fresh Fish	20 °C	75.56% (68/90)	60.00% (27/45)
	24 °C	66.67% (60/90)	53.33% (24/45)
	28 °C	48.89% (44/90)	60.00% (27/45)
	32 °C	66.67% (60/90)	31.11% (14/45)

Note: Values are pooled survival rates and quantities calculated from the total number of surviving crabs in each treatment. Juvenile treatments initially contained 90 crabs per treatment, and sub-adult treatments initially contained 45 crabs per treatment. Survival data are presented descriptively because replicate-level survival rates were not available.

## Data Availability

The data supporting the findings of this study are available from the corresponding author upon reasonable request.

## Supplementary Materials

The following supporting information can be downloaded at: https://www.mdpi.com/article/10.3390/ani16193141/s1, Table S1: Body weight, carapace length and carapace width of juvenile Chinese mitten crab (*Eriocheir sinensis*) at week 0 and week 4; Table S2: Effects of feeding regime and water temperature on growth performance parameters of juvenile Chinese mitten crab (*Eriocheir sinensis*) after the 4-week culture period; Table S3: Body weight, carapace length and carapace width of sub-adult Chinese mitten crab (*Eriocheir sinensis*) at week 0 and week 4; Table S4: Effects of feeding regime and water temperature on growth performance parameters of sub-adult Chinese mitten crab (*Eriocheir sinensis*) after the 4-week culture period; Table S5: P-values for the effects of feeding regime, water temperature, and their interaction on growth traits in juvenile and sub-adult Chinese mitten crab (*Eriocheir sinensis*) based on two-way ANOVA; Table S6: P-values of fixed effects of feeding regime, temperature, time, and their interactions on ADFR in juvenile and sub-adult Chinese mitten crab (*Eriocheir sinensis*) based on linear mixed-effects models (LMMs); Table S7: P-values of fixed effects for feeding regime, temperature, time, and their interactions on tissue-specific enzyme activities in juvenile and sub-adult Chinese mitten crab (*Eriocheir sinensis*) based on linear mixed-effects models (LMMs); Table S8: Summary of fixed-effect tests from linear mixed-effects models for apparent feed disappearance rate (ADFR) in juvenile and sub-adult Chinese mitten crab (*Eriocheir sinensis*), including F-values and degrees of freedom; Table S9: Complete fixed-effect tests of linear mixed-effects models for tissue-specific enzyme activities in juvenile and sub-adult Chinese mitten crab (*Eriocheir sinensis*), including F-values and degrees of freedom.
