# Supplementary material for: Water Temperature and Practical Feeding Regimes Interact to Affect Growth, Enzyme Activities, and Histological Characteristics in Juvenile and Sub-Adult Chinese Mitten Crab (Eriocheir sinensis)
Source: Animals (Basel). 2026 Oct 7;16(19):3141. doi: 10.3390/ani16193141 (PMC13663415; doi:10.3390/ani16193141)
Supplement: Supplementary file 1 [file animals-16-03141-s001.zip › Supplementary Tables S1-S7.pdf]

Supplementary Tables

Table S1. Body weight, carapace length and carapace width of juvenile Chinese mitten crab (*Eriocheir sinensis*) at week 0 and week 4.

Water Temperature	Time	Compound Feed			Iced Fresh Fish		
		**Weight (g)**	**Carapace Length (cm)**	**Carapace Width (cm)**	**Weight (g)**	**Carapace Length (cm)**	**Carapace Width (cm)**
20℃	Week0	16.52 ± 0.67	3.09 ± 0.04	3.34 ± 0.06	12.62 ± 0.65	2.78 ± 0.05	3.03 ± 0.05
	Week4	20.79 ± 1.04	3.28 ± 0.04	3.60 ± 0.04	16.52 ± 1.40	3.04 ± 0.02	3.32 ± 0.02
	Increment	4.24 ± 1.24[ab]	0.19 ± 0.06[bc]	0.26 ± 0.07[ab]	3.90 ± 0.76[ab]	0.26 ± 0.05[ab]	0.29 ± 0.05[a]
24℃	Week0	15.26 ± 0.84	2.96 ± 0.06	3.22 ± 0.07	14.45 ± 0.56	2.92 ± 0.04	3.20 ± 0.04
	Week4	18.75 ± 1.21	3.15 ± 0.07	3.44 ± 0.08	17.63 ± 0.51	3.14 ± 0.03	3.40 ± 0.03
	Increment	3.49 ± 1.48[bc]	0.19 ± 0.10[bc]	0.22 ± 0.10[bc]	3.18 ± 0.76[bc]	0.22 ± 0.05[bc]	0.20 ± 0.06[bc]
28℃	Week0	13.60 ± 0.65	2.84 ± 0.05	3.11 ± 0.05	10.24 ± 0.42	2.59 ± 0.04	2.84 ± 0.04
	Week4	19.96 ± 0.46	3.21 ± 0.02	3.41 ± 0.01	12.53 ± 0.25	2.68 ± 0.02	2.89 ± 0.02
	Increment	6.37 ± 0.79[a]	0.37 ± 0.05[a]	0.30 ± 0.05[a]	2.29 ± 0.49[bc]	0.09 ± 0.04[c]	0.05 ± 0.04[c]
32℃	Week0	12.07 ± 0.55	2.74 ± 0.05	2.99 ± 0.05	9.49 ± 0.54	2.51 ± 0.05	2.76 ± 0.05
	Week4	14.62 ± 0.80	2.91 ± 0.05	3.16 ± 0.05	10.90 ± 0.43	2.63 ± 0.03	2.79 ± 0.03

Water Temperature	Time	Compound Feed			Iced Fresh Fish		
		Weight (g)	Carapace Length (cm)	Carapace Width (cm)	Weight (g)	Carapace Length (cm)	Carapace Width (cm)
	Increment	2.55 ± 0.97^{bc}	0.17 ± 0.07^{bc}	0.17 ± 0.07^{bc}	1.41 ± 0.69^{c}	0.12 ± 0.06^{bc}	0.04 ± 0.03^{c}

Notes： Data are expressed as mean ± SE of three replicate tanks (n = 3). Values from crabs within the same tank were averaged before analysis. Different lowercase letters indicate that there are significant differences in the growth of body weight, carapace length and carapace width under different feeding regimes and temperature combinations. (Bonferroni-adjusted pairwise comparisons, $P < 0.05$)

Table S2. Effects of feeding regime and water temperature on growth performance parameters of juvenile Chinese mitten crab (*Eriocheir sinensis*) after the 4-week culture period.

Water Temperature	Compound Feed				Iced Fresh Fish			
	WGR(%)	SGR(%/day)	Relative carapace length change (%)	Relative carapace width change (%)	WGR(%)	SGR(%/day)	Relative carapace length change (%)	Relative carapace width change (%)
20℃	25.85 ± 8.10^{ab}	0.82 ± 0.23^{ab}	6.15 ± 1.89^{b}	7.78 ± 2.28^{a}	30.90 ± 12.98^{ab}	0.96 ± 0.35^{ab}	9.35 ± 2.09^{a}	9.57 ± 1.92^{a}
24℃	22.87 ± 10.42^{ab}	0.74 ± 0.30^{ab}	6.42 ± 3.20^{b}	6.83 ± 3.40^{a}	22.01 ± 5.90^{ab}	0.71 ± 0.17^{ab}	7.53 ± 1.80^{a}	6.25 ± 1.63^{ab}
28℃	46.76 ± 7.79^{a}	1.37 ± 0.19^{a}	13.03 ± 2.11^{a}	9.65 ± 1.79^{a}	22.36 ± 5.58^{a}	0.72 ± 0.16^{a}	3.47 ± 1.77^{a}	1.76 ± 1.60^{b}
32℃	21.13 ± 8.63^{b}	0.68 ± 0.25^{b}	6.20 ± 2.66^{b}	5.69 ± 2.43^{a}	14.86 ± 7.95^{b}	0.49 ± 0.25^{b}	4.78 ± 2.40^{a}	1.09 ± 2.13^{b}

Notes: Data are expressed as mean ± SE. WGR: weight gain rate; SGR: specific growth rate. Different lowercase letters indicate that there are significant differences in WGR, SGR, relative carapace length change and relative carapace width change under different feeding regimes and

temperature combinations. (Bonferroni-adjusted pairwise comparisons, $P < 0.05$)

Table S3. Body weight, carapace length and carapace width of sub-adult Chinese mitten crab (*Eriocheir sinensis*) at week 0 and week 4.

Water Temperature	Time	Compound Feed			Iced Fresh Fish		
		**Weight (g)**	**Carapace Length (cm)**	**Carapace Width (cm)**	**Weight (g)**	**Carapace Length (cm)**	**Carapace Width (cm)**
20°C	Week0	62.86 ± 2.11	4.89 ± 0.06	5.27 ± 0.05	54.31 ± 2.60	4.70 ± 0.09	5.10 ± 0.09
	Week4	65.12 ± 2.43	4.96 ± 0.06	5.34 ± 0.08	61.25 ± 2.03	4.85 ± 0.06	5.22 ± 0.07
	Increment	2.26 ± 2.18^{c}	0.07 ± 0.06^{bc}	0.07 ± 0.06^{b}	6.94 ± 2.70^{ab}	0.15 ± 0.09^{abc}	0.12 ± 0.09^{b}
24°C	Week0	56.48 ± 2.42	4.73 ± 0.07	5.16 ± 0.07	57.58 ± 2.16	4.81 ± 0.06	5.18 ± 0.07
	Week4	65.63 ± 2.15	4.91 ± 0.06	5.25 ± 0.05	60.73 ± 1.88	4.83 ± 0.06	5.20 ± 0.05
	Increment	9.15 ± 2.48^{a}	0.18 ± 0.08^{ab}	0.09 ± 0.08^{b}	3.15 ± 2.23^{bc}	0.02 ± 0.07^{c}	0.02 ± 0.07^{b}
28°C	Week0	61.20 ± 2.55	4.90 ± 0.07	5.27 ± 0.08	51.21 ± 2.11	4.66 ± 0.07	4.98 ± 0.07
	Week4	67.85 ± 2.89	4.92 ± 0.07	5.39 ± 0.07	62.31 ± 2.23	4.87 ± 0.07	5.27 ± 0.06
	Increment	6.65 ± 2.59^{ab}	0.02 ± 0.07^{c}	0.12 ± 0.07^{b}	11.1 ± 2.28^{a}	0.21 ± 0.07^{a}	0.29 ± 0.07^{a}
32°C	Week0	59.58 ± 2.33	4.86 ± 0.07	5.22 ± 0.07	55.11 ± 3.15	4.71 ± 0.08	5.08 ± 0.09
	Week4	66.74 ± 3.28	5.03 ± 0.08	5.34 ± 0.09	57.69 ± 1.05	4.73 ± 0.03	5.10 ± 0.02

Water Temperature	Time	Compound Feed			Iced Fresh Fish		
		Weight (g)	Carapace Length (cm)	Carapace Width (cm)	Weight (g)	Carapace Length (cm)	Carapace Width (cm)
	Increment	7.18 ± 2.42[ab]	0.17 ± 0.07[ab]	0.12 ± 0.07[b]	2.58 ± 3.19[c]	0.02 ± 0.09[c]	0.02 ± 0.09[b]

Notes：Data are expressed as mean ± SE of three replicate tanks (n = 3). Values from crabs within the same tank were averaged before analysis. Different lowercase letters indicate that there are significant differences in the growth of body weight, carapace length and carapace width under different feeding regimes and temperature combinations. (Bonferroni-adjusted pairwise comparisons, $P < 0.05$)

Table S4. Effects of feeding regime and water temperature on growth performance parameters of sub-adult Chinese mitten crab (*Eriocheir sinensis*) after the 4-week culture period.

Water Temperature	Compound Feed				Iced Fresh Fish			
	WGR(%)	SGR(%/day)	Relative carapace length change (%)	Relative carapace width change (%)	WGR(%)	SGR(%/day)	Relative carapace length change (%)	Relative carapace width change (%)
20℃	3.60 ± 5.20[a]	0.13 ± 0.18[a]	1.43 ± 1.75[a]	1.33 ± 1.80[a]	12.78 ± 6.57[ab]	0.43 ± 0.21[ab]	3.19 ± 2.35[a]	2.35 ± 2.27[a]
24℃	16.20 ± 6.27[a]	0.54 ± 0.19[a]	3.81 ± 1.99[a]	1.74 ± 1.69[a]	5.47 ± 5.13[b]	0.19 ± 0.17[b]	0.42 ± 1.77[a]	0.39 ± 1.66[a]
28℃	10.87 ± 6.61[a]	0.37 ± 0.21[a]	0.41 ± 2.02[a]	2.28 ± 2.04[a]	21.68 ± 6.64[a]	0.70 ± 0.19[a]	4.51 ± 2.17[a]	5.82 ± 1.91[a]
32℃	12.02 ± 7.04[a]	0.41 ± 0.22[a]	3.50 ± 2.22[a]	2.30 ± 2.20[a]	4.68 ± 6.28[b]	0.16 ± 0.21[b]	0.42 ± 1.82[a]	0.39 ± 1.82[a]

Notes: Data are expressed as mean ± SE. WGR: weight gain rate; SGR: specific growth rate. Different lowercase letters indicate that there are significant differences in WGR, SGR, relative carapace length change and relative carapace width change under different feeding regimes and temperature combinations. (Bonferroni-adjusted pairwise comparisons, $P < 0.05$)

Table S5. P-values for the effects of feeding regime, water temperature, and their interaction on growth traits in juvenile and sub-adult Chinese mitten crab (*Eriocheir sinensis*) based on two-way ANOVA

Stage	Indicator	feeding regime		temperature		feeding regime×temperature	
		**P-value**	**Partial eta squared（ηp²）**	**P-value**	**Partial eta squared（ηp²）**	**P-value**	**Partial eta squared（ηp²）**
Juvenile	Body weight gain	<0.001	0.538	<0.001	0.632	0.008	0.514
	Carapace length increment	0.038	0.243	0.161	0.269	0.001	0.623
	Carapace width increment	0.002	0.445	0.002	0.586	0.007	0.522
	WGR	0.086	0.173	0.024	0.437	0.057	0.367
	SGR	0.087	0.172	0.027	0.427	0.069	0.350
	Relative carapace length change	0.093	0.166	0.215	0.238	0.001	0.627
	Relative carapace width change	0.007	0.378	0.007	0.522	0.008	0.513
Sub-adult	Body weight gain	0.726	0.008	0.037	0.402	0.002	0.591
	Carapace length increment	0.750	0.007	0.966	0.016	0.002	0.596
	Carapace width increment	0.691	0.010	0.014	0.475	0.027	0.428

WGR	0.900	0.001	0.102	0.314	0.006	0.533
SGR	0.927	0.001	0.132	0.289	0.013	0.478
Relative carapace length change	0.856	0.002	0.975	0.013	0.012	0.483
Relative carapace width change	0.686	0.010	0.065	0.355	0.098	0.318

Notes: Values represent P-values from two-way ANOVA. The effects of feeding regime, water temperature, and their interaction were tested separately for each growth trait within each developmental stage. *P* < 0.05 was considered statistically significant. Mean values are shown with their corresponding 95% confidence intervals (95% CI).

Table S6. P-values of fixed effects of feeding regime, temperature, time, and their interactions on ADFR in juvenile and sub-adult Chinese mitten crab (*Eriocheir sinensis*) based on linear mixed-effects models (LMMs).

**Stage**	**Pfeeding regime**	**Ptemperature**	**Ptime**	**Pfeeding regime ×temperature**	**Pfeeding regime ×time**	**Ptemperature ×time**	**Pfeeding regime ×temperature×time**
Juvenile	<0.001	<0.001	<0.001	<0.001	<0.001	<0.001	<0.001
Sub-adult	<0.001	0.655	<0.001	0.450	<0.001	<0.001	<0.001

Notes: Values represent P-values for the fixed effects and interaction terms from linear mixed-effects models. Feeding regime, temperature, time, and their interactions were included as fixed effects, with tank included as a random effect. *P* < 0.05 was considered statistically significant. Mean values are shown with their corresponding 95% confidence intervals (95% CI).

Table S7. P-values of fixed effects for feeding regime, temperature, time, and their interactions on tissue-specific enzyme activities in juvenile and sub-adult Chinese mitten crab (*Eriocheir sinensis*) based on linear mixed-effects models (LMMs).

Stage	Tissue	Enzyme	Pfeeding regime	Ptemperature	Ptime	Pfeeding regime ×temperature	Pfeeding regime ×time	Ptemperature ×time	Pfeeding regime×temperature×time
Juvenile	Hepatopancreas	Amylase	0.065	0.892	<0.001	0.858	0.008	0.954	0.145
	Intestine	Amylase	0.127	0.591	0.244	0.623	0.174	0.634	0.513
	Hepatopancreas	Lipase	0.057	0.036	0.002	0.320	0.056	0.052	0.097
	Intestine	Lipase	0.556	0.209	0.113	0.335	0.855	0.079	0.105
	Hepatopancreas	SOD	0.285	0.450	<0.001	0.044	0.440	0.062	0.122
	Intestine	SOD	0.642	0.006	<0.001	0.035	<0.001	<0.001	0.011
	Gills	SOD	0.618	0.694	<0.001	0.398	0.764	0.854	0.834
	Muscles	SOD	0.932	0.579	<0.001	0.446	0.083	0.476	0.681
	Hepatopancreas	LDH	0.272	0.002	<0.001	<0.001	0.425	<0.001	<0.001
	Gills	LDH	<0.001	0.144	0.015	0.050	<0.001	0.316	0.157
	Muscles	LDH	0.683	0.023	0.052	0.781	0.603	0.079	0.934
	Hepatopancreas	$Na^{+}K^{+}$-ATPase	<0.001	<0.001	<0.001	0.004	<0.001	<0.001	<0.001
	Muscles	$Na^{+}K^{+}$-ATPase	0.325	<0.001	<0.001	0.005	0.472	<0.001	0.067
	Hepatopancreas	$Ca^{2+}Mg^{2+}$-ATPase	0.138	0.004	<0.001	0.082	0.054	<0.001	0.003
	Muscles	$Ca^{2+}Mg^{2+}$-ATPase	<0.001	0.003	<0.001	<0.001	<0.001	<0.001	<0.001
	Hepatopancreas	POX	0.306	<0.001	<0.001	0.005	0.609	<0.001	<0.001
	Gills	POX	0.229	0.160	<0.001	0.403	0.027	0.141	0.138

Sub-adult	Hepatopancreas	Amylase	0.873	0.859	<0.001	0.727	0.003	0.709	0.078
	Intestine	Amylase	0.680	0.506	<0.001	0.883	0.848	0.661	0.355
	Hepatopancreas	Lipase	0.063	0.729	0.009	0.554	0.099	0.295	0.233
	Intestine	Lipase	0.006	0.072	<0.001	0.684	<0.001	0.054	0.001
	Hepatopancreas	SOD	0.829	0.671	0.002	0.930	0.043	0.389	0.414
	Intestine	SOD	<0.001	0.657	<0.001	0.034	0.002	0.116	0.007
	Gills	SOD	0.340	0.490	0.006	0.510	0.119	0.367	0.083
	Muscles	SOD	0.819	0.995	0.005	0.452	0.464	0.895	0.110
	Hepatopancreas	LDH	0.019	0.003	<0.001	0.535	<0.001	<0.001	0.031
	Gills	LDH	0.298	0.069	<0.001	0.148	0.367	0.003	0.104
	Muscles	LDH	0.541	0.732	<0.001	0.837	0.082	0.093	0.013
	Hepatopancreas	Na^+K^+-ATPase	0.003	0.322	<0.001	0.210	<0.001	0.010	0.002
	Muscles	Na^+K^+-ATPase	0.060	0.249	0.046	0.493	0.290	0.007	0.055
	Hepatopancreas	$Ca^{2+}Mg^{2+}$-ATPase	<0.001	0.032	<0.001	0.832	<0.001	0.290	0.867
	Muscles	$Ca^{2+}Mg^{2+}$-ATPase	0.018	0.001	<0.001	0.007	0.351	<0.001	<0.001
	Hepatopancreas	POX	<0.001	<0.001	<0.001	0.001	<0.001	<0.001	<0.001
	Gills	POX	<0.001	<0.001	<0.001	<0.001	<0.001	<0.001	<0.001

Notes: Values represent P-values for each fixed effect and interaction term derived from LMMs. Fixed effects included feeding regime, temperature, time, and their two-way and three-way interactions. The random structure included a random intercept for tank to account for repeated observations within experimental tanks. Mean values are shown with their corresponding 95% confidence intervals (95% CI).
